# Spatiotemporal Stability of Neonatal Rat Cardiomyocyte Monolayers Spontaneous Activity Is Dependent on the Culture Substrate

**DOI:** 10.1371/journal.pone.0127977

**Published:** 2015-06-02

**Authors:** Jonathan Boudreau-Béland, James Elber Duverger, Estelle Petitjean, Ange Maguy, Jonathan Ledoux, Philippe Comtois

**Affiliations:** 1 Research Centre, Montreal Heart Institute, Montreal, H1T 1C8, Quebec, Canada; 2 Department of Molecular and Integrative Physiology, Université de Montréal, Montreal, H3T 1J4, Quebec, Canada; 3 Institute of Biomedical Engineering, Université de Montréal, Montreal, H3T 1J4, Quebec, Canada; 4 Department of Medicine, Université de Montréal, Montreal, H3T 1J4, Quebec, Canada; Centro Cardiologico Monzino, ITALY

## Abstract

In native conditions, cardiac cells must continuously comply with diverse stimuli necessitating a perpetual adaptation. Polydimethylsiloxane (PDMS) is commonly used in cell culture to study cellular response to changes in the mechanical environment. The aim of this study was to evaluate the impact of using PDMS substrates on the properties of spontaneous activity of cardiomyocyte monolayer cultures. We compared PDMS to the gold standard normally used in culture: a glass substrate. Although mean frequency of spontaneous activity remained unaltered, incidence of reentrant activity was significantly higher in samples cultured on glass compared to PDMS substrates. Higher spatial and temporal instability of the spontaneous rate activation was found when cardiomyocytes were cultured on PDMS, and correlated with decreased connexin-43 and increased CaV3.1 and HCN2 mRNA levels. Compared to cultures on glass, cultures on PDMS were associated with the strongest response to isoproterenol and acetylcholine. These results reveal the importance of carefully selecting the culture substrate for studies involving mechanical stimulation, especially for tissue engineering or pharmacological high-throughput screening of cardiac tissue analog.

## Introduction

Cardiomyocytes are central to the electromechanical properties of the heart. Cardiac tissue can exhibit sensitivity to arrhythmias, which are complex spatiotemporal electrical activities. Dynamic electrical behaviors of cardiac tissue include normal propagation from pacemaker sites [1, 2], stable rotating spiral waves [3], unstable spiral waves that break up during propagation [4], and bursts of activity often associated with the initiation and termination of spiral waves [5]. *In situ*, cardiomyocytes are electrically excitable; some exhibit spontaneous activity associated with low contractility while most working cardiomyocytes show no spontaneous activity [6]. An increase in membrane potential (diastolic depolarization) above threshold initiates an action potential through the sequential opening and closing of membrane ionic channels, generating membrane currents that trigger contraction of the cell [7].

Sympathetic and parasympathetic stimulation are among the main mechanisms through which the autonomic nervous system controls heart rate; for example, β-adrenergic stimulation positively shifts voltage dependence of the hyperpolarization-activated cyclic nucleotide-gated (HCN) channels that results in an increased current density [8] and faster diastolic depolarization.

Spontaneous activity can be physiologically necessary (sinoatrial node as the primary pacemaker site, atrioventricular node, and Purkinje fibers) [6] or detrimental (ectopic activity leading to arrhythmia genesis) [9]. Spontaneous activity, designated the “membrane” or “voltage” clock, is the result of a delicate balance between inward and outward currents resulting from the activity of several voltage-dependent ion channels [6]. HCN forming the funny current (*I*_f_), L-type Ca^2+^ channels, T-type Ca^2+^ channels, and delayed rectifier K^+^ channels are among the ion channels expressed at the cardiomyocyte plasma membrane that contribute most to the membrane clock [10]. *I*_f_ is believed to be a key player in pacemaker driving capabilities [11]. In the intracellular space, Ca^2+^ cycling contributes to activation through Ca^2+^ release and reuptake from the sarcoplasmic reticulum and membrane flux via the Na^+^/Ca^2+^ exchanger, and has been designated the “calcium” clock [12].

Excitation—contraction coupling links the electrical excitation of cardiomyocytes to cellular contraction [13]. There is also a feedback mechanism, termed mechano-electrical feedback (MEF), which connects mechanical constraints to cardiac electrical activity [14]. MEF has been shown to modulate the rate of spontaneous activity of the sinoatrial node [15] and the conduction of electrical impulses [16], thus promoting complex spatial activity. *In vivo*, the microenvironment surrounding cells is composed of a mixture of heterogeneous physical, chemical, and mechanical properties. Modification of these parameters impacts cell fate by controlling multiple aspects of their behavior, including growth, proliferation, differentiation, migration, and gene expression [17–19], as well as regulating functional properties of cardiomyocytes [20–22], such as contractility. Cardiomyocyte spontaneous activity is dependent on the expression of ion channels that can be modified under many conditions, such as altering the mechanical properties of the cell microenvironment [22–24], electrical stimuli [25], and mechanical stimuli [26]. Integration of these properties in a culture environment may lead to the ability to control the spontaneous rate of bioengineered cells.

Cell culture of cardiac tissue analog is becoming increasingly interesting for regenerative medicine (conditioning of pre-injected stem cell—derived cardiomyocytes [20, 27] and tissue engineering [28–32]) and high throughput for cardiotoxicity evaluation [33, 34]. *In vitro*, rhythmic contraction can be sustained for days when cardiomyocytes are grown on hard, ligand-coated culture materials (e.g. glass and polystyrene); however, cells ultimately lose their rhythmic contractions and myofibrils [35]. Cardiomyocytes are known to have stretch and stress sensors responsive to changes in myocardial mechanical conditions occurring in pathology or aging [21]. Currently, most mechanical stimulation experiments use the deformation of an elastic substrate (e.g. Flexcell) [36–40]. Adequate understanding of the impact of the elastic substrate on which cells are to be cultured for mechanical stimulation is an important step towards optimizing culture conditions. Many studies have shown an important effect of substrate stiffness on cell function, notably that a substrate with tissue-like stiffness is most appropriate to optimize cardiomyocyte contraction force [41]. It has also been proposed that stiffness influences the rate of spontaneous activity of stem cell—derived cardiomyocytes [42]. Interestingly, myocardial conduction is significantly optimized when the stiffness of the cell culture environment matches that of cardiac cells [43]. Different elastic substrates have been used to study the effect of mechanical properties (microenvironment or deformation) on cells; these materials include polydimethylsiloxane (PDMS), polyacrylamide, alginate, chitosan, agarose, and polyesters [44]. Silicone-based materials offer better tolerance to mechanical deformation compared to hydrogels like polyacrylamide that are weak and brittle; indeed, polyacrylamide is not a suitable material for stretch-related studies [45]. PDMS is hydrophobic and does not allow direct cell attachment and proliferation [46]. This issue can be resolved by coating PDMS with proteins that attach to hydrophobic surfaces, such as gelatin or fibronectin [46]. In addition to surface coating, PDMS can be temporarily rendered hydrophilic by exposing the surface to air plasma [46].

In the present study, we chose to investigate the impact of substrate type (glass vs PDMS) on the frequency, stability, and organization of spontaneous activity, as well as the impact of sympathetic and parasympathetic stimulation, on neonatal rat cardiomyocyte monolayers. We also evaluated the level of different genes implicated in the generation of action potentials and electrical conduction mechanisms. As such, the expression of HCN2 and CaV3.1 mRNA increased on the softer substrate. When focusing on autonomous activity, we observed an increased number of activation sites (meaning changes in the location of activation) on PDMS compared to the glass substrate. This was related to an increased instability of spontaneous activity and a tendency towards a decreased expression of connexin-43 (Cx43) on the PDMS substrate. Finally, cardiomyocytes cultured on PDMS 1:20 exhibited the greatest increase in the frequency of contraction after stimulation with ISO and the greatest decrease with ACh compared to cultures on glass. This study highlights the importance of carefully evaluating the choice of elastic substrate for mechanical stimulation. Silicone-based PDMS can favor unstable spontaneous rhythms and responses to pharmacological stimuli by altering intercellular connectivity and ion channels influencing the voltage clock of cardiomyocytes.

## Materials and Methods

All animal-handling procedures were concordant with the Canadian Council on Animal Care guidelines and approved by the institutional Animal Research Ethics Committee (*Permit Number*: *2011-35-01P*). Efforts were made to minimize suffering.

### PDMS substrate fabrication

Polydimethylsiloxane (*PDMS*, *SYLGARD 184*, *Dow Corning*) is a binary compound chosen because of its elasticity and optical transparency. Mass ratio of pre-polymer (base) and cross-linker (curing agent) can be varied to get a softer to harder material after 48 hours of curing at room temperature [47–49]. De-foaming is performed to remove air bubbles and spin coating (2100 rpm) for 10 seconds is applied to get a thin and uniform surface. The calculated thickness of the PDMS layer is approximately 200 μm. After full curing of the PDMS, but just before coating and seeding of the cells, the dishes were plasma-cleaned (*Harrick Plasma*, *PDC-32G*) to increase the wettability of the surface [50]. Different substrates were used: glass, PDMS ratio (curing agent:base) of 1:20, and PDMS ratio of 1:40. These PDMS ratios were chosen because they are close to the 1:10 ratio suggested by the manufacturer while having Young’s moduli close to the physiological value.

### Cell isolation and culture

Isolation was performed according to the protocol of the neonatal cardiomyocyte isolation kit (*Worthington*, *Lakewood*, *NJ*, *USA*). Briefly, rats aged 1‒3 days old (*Sprague-Dawley*, *Charles River Laboratories*, *Saint-Constant*, *QC*, *Canada*) were sacrificed by decapitation. Hearts were rapidly excised and immediately placed in cold Ca^2+^- and Mg^2+^-free Hank’s Balanced Salt Solution. Ventricular muscle was harvested and then minced on ice into 1‒3 mm^3^ pieces. Purified enzymatic digestion (50 μg/mL trypsin and 136 μg/mL collagenase) was then used to gently dissociate cardiomyocytes. Isolated cells (enriched cardiomyocytes) were counted and seeded at a density of 10^6^ cells/mL in 20-mm diameter glass-bottom culture dishes (*D29-20-0-N*, *In Vitro Scientific*, *Sunnyvale*, *CA*, *USA*) pre-coated (on glass or on PDMS) with a mixture of 0.2% porcine-derived gelatin (*G1890*, *Sigma-Aldrich*, *Oakville*, *Ontario*, *Canada*) and 0.00125% fibronectin solution (*F1141*, *Sigma-Aldrich)* [51]. Cells were grown for 24 hours in DMEM (*319-050-CL*, *Wisent Inc*., *St-Bruno*, *Canada*) with 5% fetal bovine serum (*SH30396*.*03*, *Fisher Scientific Co*. *Ltd*, *Ottawa*, *Ontario*, *Canada*) and 1% penicillin/streptomycin (*450-201-EL*, *Wisent Inc*.). Cardiomyocytes were then starved of fetal bovine serum for another 24 hours in DMEM with 1% penicillin/streptomycin prior to experimentation.

The specific methodology for cell population evaluation based on gene expression can be found in S1 Appendix.

### Spontaneous contraction recordings

Phase contrast images of neonatal cardiomyocytes were acquired 48 hours post seeding *in vitro* with a Dalsa HM640 camera (60 frames by second) coupled to an inverted Nikon optical microscope (10X magnification and a field of view of 0.34 mm × 0.45 mm). Spontaneous activity measurements were performed with an algorithm developed in Matlab. Video analysis was carried out as follows: a frame of reference corresponding to the pre-contraction state of the cells (M_rest_) is determined and the corresponding 2D image is then subtracted to all acquired frames (M_diff_(x,y,t) = M(x,y,t)—M_rest_). The sum of the absolute difference M_diff_(x,y,t) over all pixels (x,y) divided by the number of pixels serves as an aggregate temporal signal. A moving window of 5 samples is then applied to smooth the signal followed by baseline drift removal (subtraction of 40 samples moving window filtered signal). Spontaneous frequency, standard deviation of period (σ_noise_), and pauses (corresponding to time interval greater than 3 seconds) were calculated from the video data. Videomicroscopy (30 seconds recordings) was performed at 37°C over a 20 minutes period at the following time points: 0 (pre-drug), 1, 5, 10, 15, and 20 minute(s) for each condition (glass, PDMS 1:20, and PDMS 1:40).

### Mapping of calcium dynamics

After 48 hours of culture, the cardiomyocytes were washed once with fresh media and incubated with 10 μM of fluo-4 AM (*F-14201*, *Life technologies*, *Burlington*, *Ontario*, *Canada*) and 0.2% Pluronic acid F-127 (*P-3000MP*, *Life Technologies*) for 30 minutes at 37°C. Fluo-4—loaded cardiomyocytes were then washed 4 times with fresh media, followed by a 15 minutes resting period to allow de-esterification of the dye before starting calcium transient mapping experiments performed in DMEM at 37°C. Fluorescence was recorded for 30 seconds at 125 Hz with a CardioCCD camera (*RedShirt Imaging*, *Decatur*, *GA*, *USA*). The dye was excited with a quartz tungsten halogen lamp (*Oriel Instruments Inc*., *Stratford*, *CT*). The filters used for excitation and emission were λ_excitation_ ≈ 480±20 nm (*Chroma Technology Corp*, *Bellows Falls*, *VT*) and λ_emission_ ≈ 535±25 nm (*Semrock Inc*., *Rochester*, *NY*), respectively. The system was set to image with an acquisition frame rate of 125 Hz for all experiments. Signals were filtered and analyzed using a program developed in-house using Matlab software (*R2008*, *MathWorks Inc*., *Natick*, *MA*). In brief, raw acquisitions were normalized by the minimum fluorescence for each pixel and the dF/dt (the first time derivative of fluorescence) was approximated using a finite difference approach. The resulting signals were then spatially filtered using a Gaussian kernel (5×5 pixels with σ = 1.5) followed by a temporal moving average with a two-sample window.

### Quantitative Polymerase Chain Reaction (qPCR)

Briefly, cultured neonatal cardiomyocytes were harvested in RA1 lysis buffer from Nucleospin RNA II kit (*Macherey Nagel GmbH & Co*. *KG*, *Waltham*, *MA*, *USA*). mRNA was isolated using the same kit following the manufacturer’s instructions, including DNAse treatment to prevent genomic contamination. mRNA was then reverse-transcribed with the High-capacity Reverse Transcription kit (*Applied Biosystems*, *Burlington*, *Ontario*, *Canada*). qPCR was performed with TaqMan probes from Applied Biosystems for housekeeping genes (hypoxanthine guanine phosphoribosyl transferase (HPRT), Glyceraldehyde 3-phosphate dehydrogenase (GAPDH), and Beta-2 microglobulin (β2M) and with primers designed for SYBR Green experiments to assess the expression of Kir2.1, CaV3.1, CaV3.2, HCN2, HCN4, Cx43, Kir3.1, Kir3.4, and Adrβ1 (S1 Table for sequences). The geometric mean of the expressions of HPRT, β2M and GAPDH was used for normalization. qPCR reactions were performed with TaqMan Gene Expression Master Mix and Power SYBR Green kits from Applied Biosystems. Reactions were run on an Mx3000 qPCR System from *Stratagene*. Relative gene expression values were calculated by the 2^-ΔCt^ method.

### Sympathetic and parasympathetic stimulation

Acute effects of the sympathetic agonist, isoproterenol (ISO, *I6504*, *Sigma-Aldrich*) and the parasympathetic agonist, acetylcholine (ACh, *A2661*, *Sigma-Aldrich*) were studied by videomicroscopy and calcium imaging at 48 hours post-culture. Final concentrations of 100 nM ISO and 1 μM ACh were added to the culture media to stimulate sympathetic and parasympathetic pathways, respectively. Drug effects were evaluated by videomicroscopy. In a second set of experiments, changes in spatiotemporal activity by ISO and ACh were studied using calcium imaging with fluo-4.

### Cell proliferation assay

Cultured neonatal rat cardiomyocytes were washed with media (*DMEM*, *Wisent*) and then fixed with 2% paraformaldehyde for 15 minutes. Cells were permeabilized in 1% bovine serum albumin solution (*Millipore*, *Etobicoke*, *Ontario*, *Canada*) with 1% triton X-100 (*TRX506*, *Bioshop*, *Burlington*, *Ontario*, *Canada*). Cardiomyocytes were then stained with 4',6-diamidino-2-phenylindole (*DAPI*, *D1306*, *Life Technologies*) (1:1000) and finally the seeding area was cut from the membrane and transposed in 20 mm diameter glass-bottom culture dishes (*D29-20-0-N*, *In Vitro Scientific*) with Mowiol mounting medium (*Mowiol 4–88*, *81381*, *Sigma-Aldrich*). Imaging was performed with a Zeiss LSM710 inverted confocal microscope.

### Statistical analysis

Data were analyzed using Prism 5 by GraphPad. One-way analysis of variance (anova) followed by Tukey multiple comparison test was used to compare groups unless otherwise stated. A p-value below 0.05 was considered statistically significant.

## Results

### Stiffness of the PDMS substrate

Stiffness measured as a function of the ratio of pre-polymer (base) and cross-linker (curing agent) is presented in supplementary data (S1 Fig). Stiffness is expressed as the modulus of elasticity measured by Young’s modulus formula (S2 Appendix). PDMS mixed in ratios of curing to base agent (curing:base) produced substrates with mean moduli of 974*±*32 kPa (1:5), 293*±*8 kPa (1:10), 112*±*6 kPa (1:15), 87*±*22 kPa (1:20), 42*±*6 kPa (1:25), 27*±*4 kPa (1:30) and 16*±*4 kPa (1:40). In comparison, stiffness of the glass surface has been shown to be ~30 GPa [22].

### Spontaneous activity in monolayer cultures

The effect of substrate stiffness on spontaneous activity was first evaluated by videomicroscopy. Softer substrate tends to diminish the autonomous contraction frequency of cultured cardiomyocytes as spontaneous frequency was decreased by 24.4% (PDMS 1:20) and 30.8% (PDMS 1:40) compared to glass (S2A Fig). Interestingly, the variability in the frequency within groups is much greater in the glass group compared to PDMS groups with a subset of higher frequencies.

**Fig 1:**
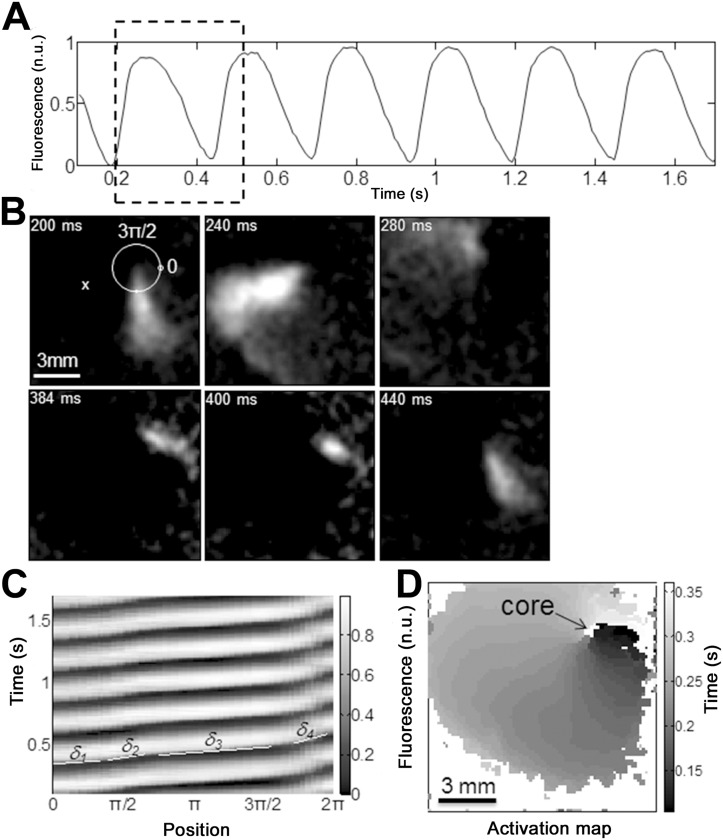
Spontaneous frequency on glass vs. PDMS substrates. Example of a spontaneous reentry as observed on glass substrate. A. Normalized calcium transients over time obtained from the pixel marked by a white × in panel B. B. Snapshots of the dF/dt over 1 rotation of reentry with time stamps indicated at the top left of each panel. The white circle in the top left snapshot at 200 ms illustrates a trajectory around the reentry core (scale bar is 3 mm) used for the time/phase plot depicted in panel C. The period of activity in this example was highly stable around 255 ms. C. Propagation along the trajectory depicted as a white circle in panel B shows periodic activity. However, it is clear that the activation delay δ (δ = slope) is not constant with large regions of rapid velocity along the trajectory from 3π/4 to 3π/2, while the region close to 2π illustrates a slow conduction area. The local activation delay was not uniform along the reentrant trajectory and changed from δ_1_ = 0.15 second/cm to δ_2_ = 0.4 second/cm, δ_3_ = 0.18 second/cm, and finally δ_4_ = 0.66 second/cm. D. Activation map obtained for the time interval between 0.1 and 0.35 second of reentrant activity. The maps shows a central core of ~0.31 mm^2^ (indicated by the arrow) and highlights the slow region of the pathway of reentry (time near 0.1 to 0.15 second on the top right of the map).

A mapping study of calcium activity was performed to determine if spatiotemporal activity could explain the presence of high-frequency activity. Although most of the activity was ectopic in nature with spatially restricted origin sites, spontaneous reentry could also be detected in the glass substrate group as illustrated with a typical example in Fig 1. The time course of normalized calcium transients (Fig 1A) was obtained from the pixel marked by an “x” (in Fig 1B). Snapshots of the dF/dt are depicted in Fig 1B over 1 rotation of reentry. The period of activity in this example was highly stable at 255 ms. Propagation along the circular trajectory (which is not always perpendicular to the activation front) depicted as a white circle (Fig 1B) shows periodic activity (Fig 1C). However, it is clear that the local activation delay δ (δ = slope) is not constant with large regions of rapid velocity along the trajectory from 3π/4 to 3π/2, while the region close to 2π illustrates a slow conduction area. Local activation delay was not uniform along the reentrant trajectory and changed from δ_1_ = 0.15 second/cm to δ_2_ = 0.4 second/cm, δ_3_ = 0.18 second/cm, and finally δ_4_ = 0.66 second/cm. The activation map (Fig 1D) was obtained for the time interval between 0.1 and 0.35 second of reentrant activity. The maps shows a central core of ~0.31 mm^2^ (indicated by the arrow) and highlights the slow region of the reentrant pathway (time near 0.1 to 0.15 second).

The high frequency of reentrant activity found in mapping led us to go back to the videomicroscopy data. Further analysis of the videomicroscopy mean frequency recorded at different time points (0, 1, 5, 10, 15, and 20 minutes) for a duration of 30 seconds shows a significantly higher incidence of high-frequency reentrant activities for cardiomyocyte monolayers cultured on glass compared to PDMS (5% for glass and 0% for both PDMS 1:20 and 1:40, p<0.05 with Fisher’s exact test when comparing samples with at least 1 episode of frequency >3 Hz). High-frequency samples were assumed to be associated with reentrant activity and appeared responsible for the frequency of substrate-dependent activity. Indeed, removal of these reentrant-associated samples (defined as frequencies >3Hz) resulted in a loss of difference, as the mean spontaneous activity was reduced by 1.5% and 9.8% for cardiomyocytes cultured on PDMS (1:20 and 1:40, respectively) compared to glass (S2B Fig). Thus, higher mean frequency of spontaneous activity of monolayers on glass detected by videomicroscopy could be explained by a greater probability of spontaneous reentry.

### Stability of the spontaneous activity

**Fig 2:**
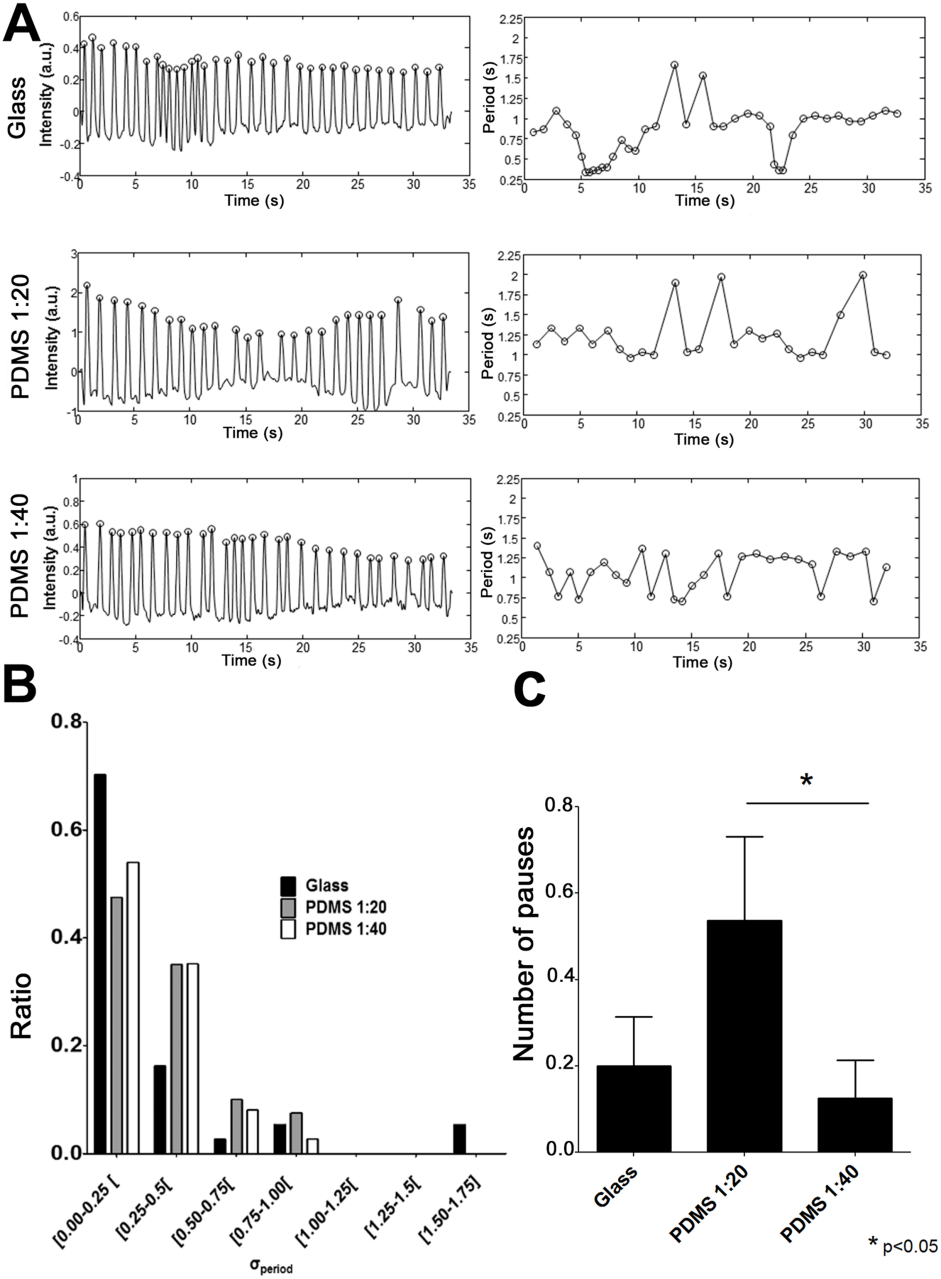
Temporal variation in the period of activity. A. Left panel shows a sample signal of spontaneous activity obtained by videomicroscopy and right panel shows the calculated interbeat period. The standard deviation of the temporal distribution of activation period (σ_period_) was calculated as an estimate of temporal variability. B. Histogram of σ_period_ obtained for the glass, PDMS 1:20, and PDMS 1:40 groups. The median of the σ_period_ calculated for each group is: 0.19 second (glass), 0.29 second (PDMS 1:20), and 0.23 second (PDMS 1:40); n = 44, N = 11.

Temporal variation of the interbeat interval is an important parameter of spontaneous activity, reflecting the stability of cardiac homeorhesis. A strong oscillator, as desired in tissue engineering aimed at the development of biopacemakers [52] should show less variability in the interbeat interval, while a weak oscillator may be more sensitive to perturbation yielding more variation in spontaneous activity. Thus, the standard variation in the interbeat interval (σ_period_) from a 30 seconds acquisition was used as a measure of temporal activity. Typical examples of peak-detection and period signatures are presented in the left column of Fig 2A and the correlated interbeat intervals (time difference between peaks highlighted by circles in the column on the left) are displayed in the columns on the right. The rate of spontaneousactivation appeared less stable with greater variance of the PDMS groups compared to glass (Fig 2B, Ansari-Bradley test, p<0.05). PDMS 1:20 had the highest variation (median σ_period_ = 0.29 second), followed by PDMS 1:40 (median σ_period_ = 0.23 second), and glass (median σ_period_ = 0.19 second). Evaluation of the number of pauses (absence of spontaneous contraction for longer than 3 seconds) shows that pauses are significantly more frequent in the PDMS 1:20 group compared with the PDMS 1:40 group (presented in Fig 2C). The ratio of samples with at least 1 pause were calculated as 0.1, 0.24, and 0.05 for glass, PDMS 1:20, and PDMS 1:40, respectively. There was a significant difference between the two PDMS groups.

### Number of activation sites

**Fig 3:**
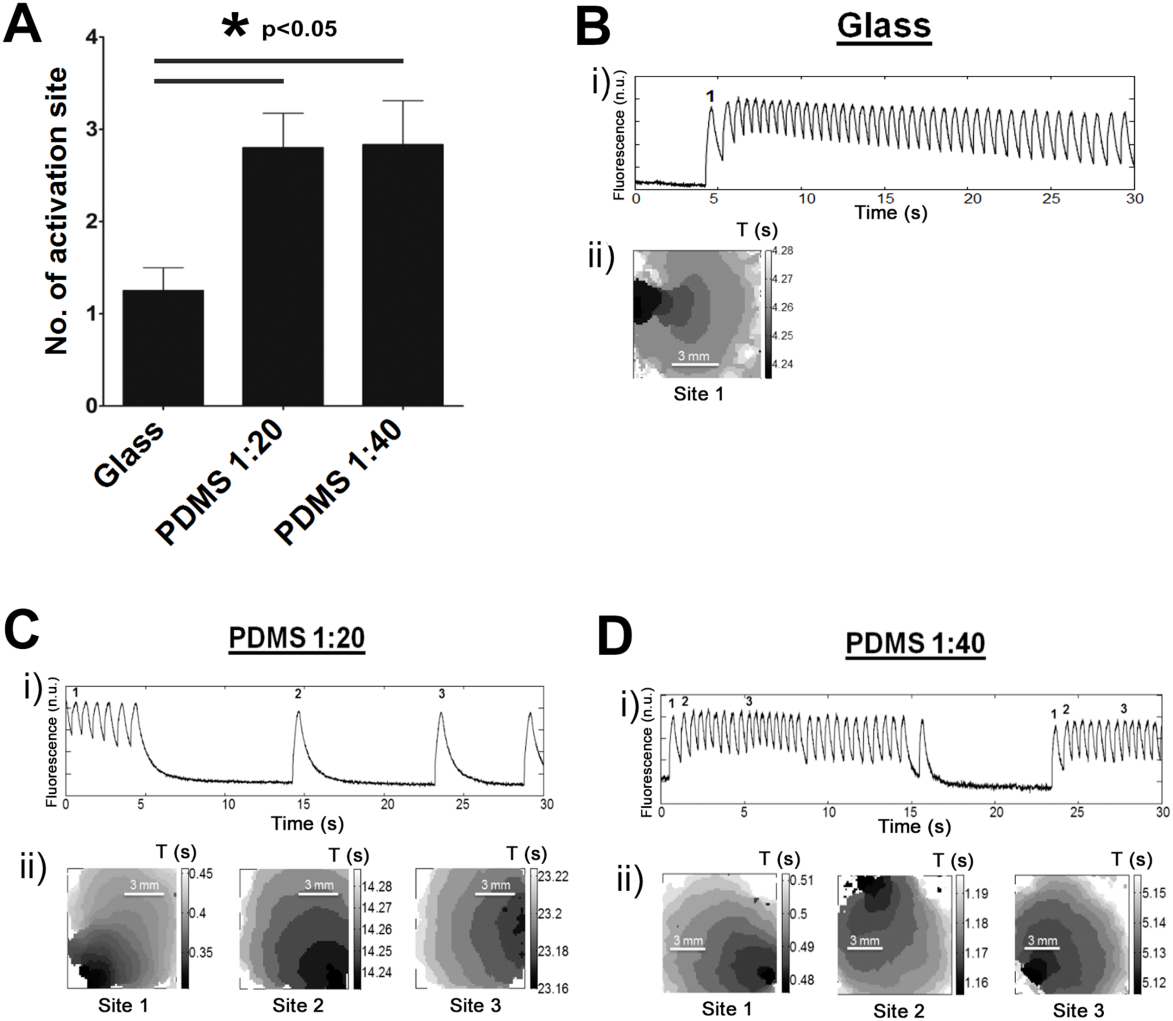
Increased number of initiation sites on PDMS substrate. A. The mean number of activation sites for cardiomyocyte monolayers cultivated on glass, PDMS 1:20, and PDMS 1:40 were 1.3±0.3, 2.8±0.4, and 2.8±0.5 sites, respectively. The mean number of activation sites is significantly higher on PDMS compared to glass (p<0.05). Examples of activation patterns on different substrates. i) A trace of calcium transients of the spontaneous activity is shown with ii) activation maps of the first beat for each different activation site for glass (B), PDMS 1:20 (C), and PDMS 1:40 substrates (D). Sites are labeled with a number on top of the calcium transient. The corresponding site number is indicated at the time it starts to drive the monolayer (calcium transients without corresponding numbers originated from the same initiation site as the previous one). In these examples, 1 initiation site was observed on glass compared to 3 sites for both PDMS substrates.

Mapping data (30 seconds acquisitions) revealed that the number of activation sites (indicative of spontaneous activity) of the entire seeding area of the petri dishes (20 mm) was generally lower for cardiomyocyte monolayers cultured on glass compared to PDMS. As shown in Fig 3A, there is a 115% increase in the number of activation sites on PDMS (2.8±0.4 sites with PDMS 1:20 and 2.8±0.5 sites with PDMS 1:40) compared to glass (1.3±0.3 sites).

**Fig 4:**
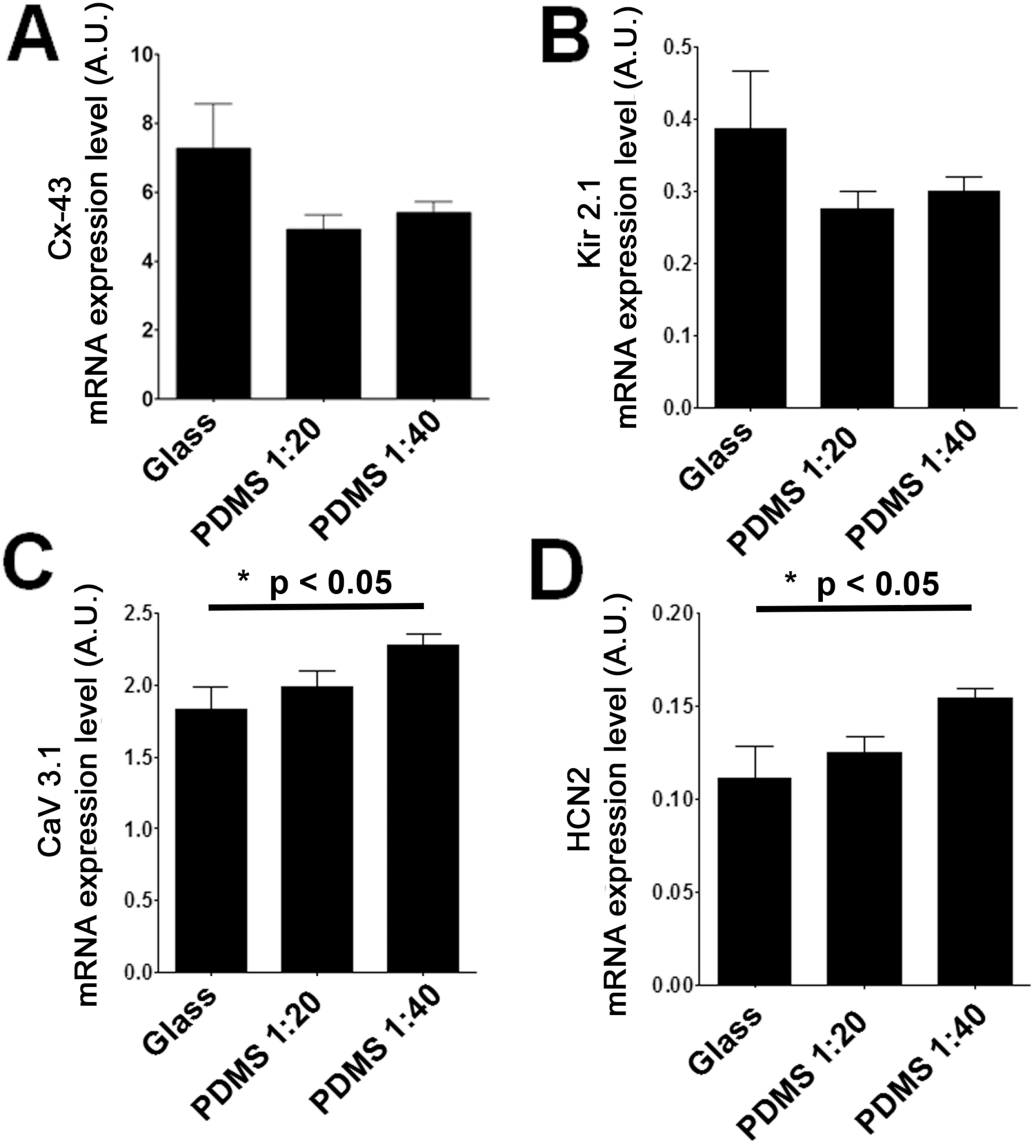
Role of the proteins expression on spontaneous activity. A. Cx43 mRNA expression was 6.24±0.89, 4.55±0.27 and 5.70±0.12. According to our results, Cx43 mRNA expression has a tendency to be lower on PDMS substrates compared to glass, however, significant differences was detected between PDMS 1:20 and PDMS 1:40. B-D. mRNA expression of targeted proteins playing a role on automaticity through the voltage clock could explain the small changes observed in rhythm. Kir2.1 (B) mRNA expression tends to be lowered when cardiomyocytes are cultivated on PDMS compared to glass. There is a tendency towards increased expression of CaV3.1 (C) and HCN2 (D) on PDMS compared to glass (p<0.05, between glass and PDMS 1:40).

Examples of activity obtained from a cardiomyocyte population cultured on different substrates are presented in Fig 3B–3D. Panel i shows a temporal trace of the activity recorded with fluo-4, while panel ii illustrates the activation map of the first beat of each different site of activation found in these examples. Mean interbeat interval of monolayers (Fig 3B) cultured on glass was 0.64±0.02 second. In the examples shown, only 1 site of activation was found (middle left side of the monolayer). For PDMS 1:20, the mean interbeat interval for the example presented in Fig 3C was 3.55±1.44 seconds. Within a 30 s recording period, a total of 3 different activation sites were found. On PDMS 1:40 (Fig 3D), the mean interbeat interval was 0.70±0.18 second and activation occurred over 3 sites. Heterogeneity in the spontaneous pacemaking activity of isolated neonatal rat cardiomyocytes is well established [53]. Intercellular communication might therefore affect automaticity of a monolayer consisting of beating and non-beating cells [54, 55]. Intercellular communication was then probed through the expression of Cx43, the main protein involved in cardiac electrical coupling. Surprisingly, a lower mRNA expression of Cx43 (p = 0.12) was noted in monolayers cultured on PDMS (1:20 or 1:40) compared with glass (Fig 4A). Decreased cellular coupling could therefore increase the propensity for the multifocal activation observed on PDMS.

### Effect of voltage clock—associated mRNA expression on spontaneous activity

The voltage clock, an essential component of cardiomyocyte excitability and spontaneous activity might also be altered by the culture substrate. Changes in voltage clock—associated gene (Kir2.1, CaV3.1, CaV3.2, HCN2, and HCN4) expression were then sought at the mRNA level by qPCR. Inward-rectifier K^+^ ion channels (Kir2.1) are key stabilizers of cardiac excitability and as expected, mRNA levels for Kir2.1 (Fig 4B) appear to be lower on PDMS (PDMS 1:20, 0.28±0.02; PDMS 1:40, 0.30±0.02) than on glass (0.39±0.08). mRNA expression was 28% and 23% lower in PDMS 1:20 and PDMS 1:40, respectively, compared to glass. T-type calcium channels (CaV3.1 and CaV3.2) are native low voltage—activated calcium channels involved in cardiac pacemaker activity [56]. CaV3.1 (Fig 4C) is significantly increased by 24% on PDMS 1:40 (2.27±0.08) compared to glass (1.83±0.16), while a non-significant increase of 9% was detected for PDMS 1:20 (1.99±0.11). In contrast, CaV3.2 (S4A Fig) decreased by 10% on PDMS 1:40 (0.21±0.02) and by 25% on PDMS 1:20 (0.17±0.01) compared to glass (0.23±0.02). HCNs form the pacemaker current (*I*_f_), known to promote spontaneous activity through its hyperpolarization-dependent activation [8]. HCN2 (Fig 4D) has an increased expression on PDMS (PDMS 1:40, 0.15±0.01) compared to glass (0.11±0.02; p<0.05), while there was no significant difference in mRNA expression of HCN4 (S4B Fig) (glass, 0.50±0.08; PDMS 1:20, 0.49±0.09; and PDMS 1:40, 0.54±0.08).

### Parasympathetic and sympathetic modulation of spontaneous activity

Relative spontaneous frequency was obtained by videomicroscopy and calculated by taking spontaneous frequency of contraction recorded at t = 1, 5, 10, 15, and 20 minute(s), and by dividing that value by the spontaneous frequency prior to the addition of the drug (pre-drug at t = 0). Appropriate controls (without drugs) showed lack of time-dependent effects as illustrated in S4C Fig

**Fig 5:**
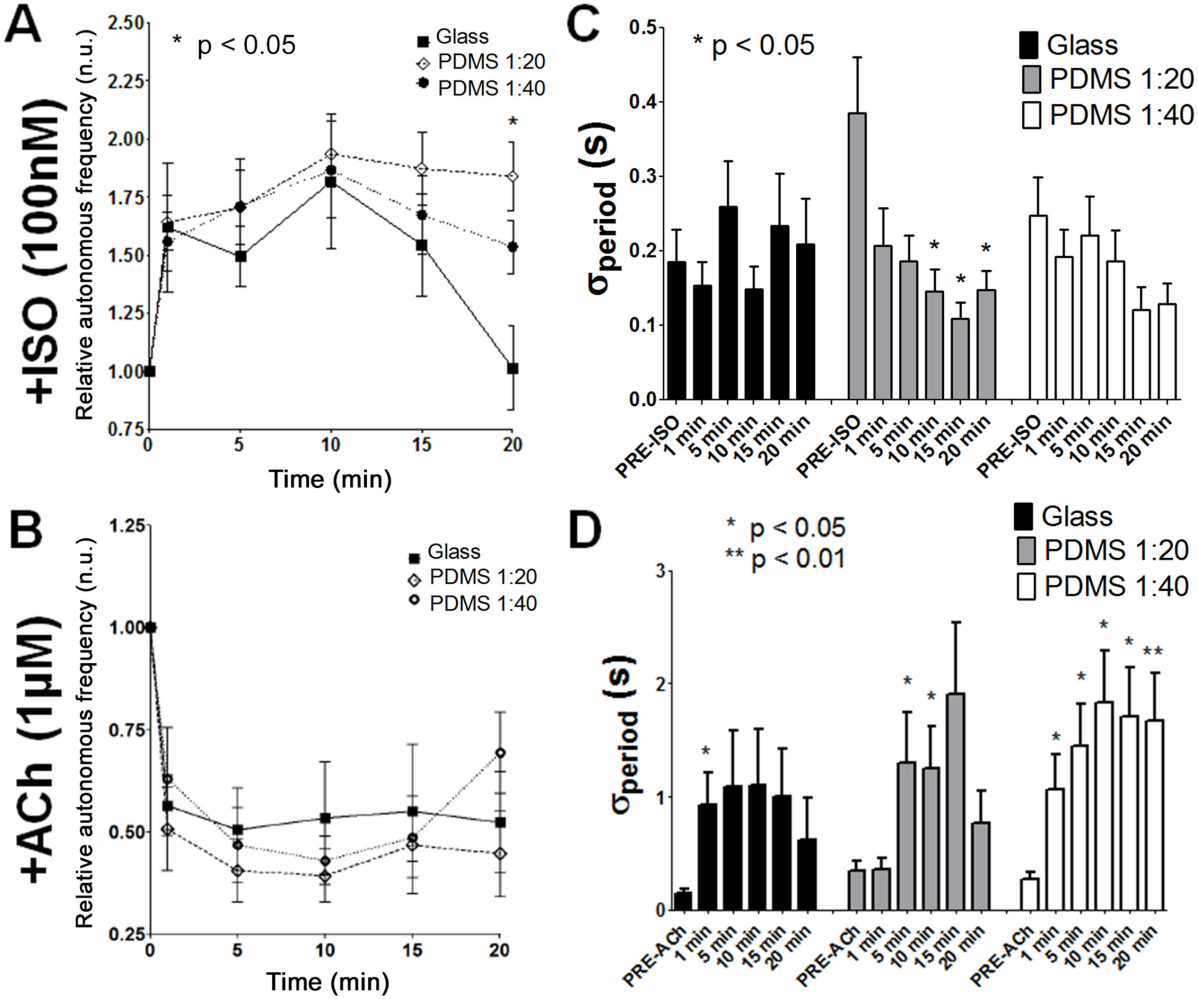
Effects of parasympathetic and sympathetic stimulation on the rate of spontaneous activity. Relative spontaneous frequency was obtained by videomicroscopy and calculated by taking spontaneous frequency recorded at t = 1, 5, 10, 15, and 20 minute(s) and by dividing that value by the spontaneous frequency prior to the addition of the drug (pre-drug at t = 0). A. Sympathetic stimulation by addition of 100 nM of ISO. The rates of contraction at t = 1 minute after the addition of ISO were 1.62±0.28 Hz (glass), 1.64±0.12 Hz (PDMS 1:20), and 1.56±0.13 Hz (PDMS 1:40); consequently, no significant differences were observed at t = 1 minute between the groups (p = 0.53). However, the rates of contraction at t = 20 minutes after the addition of ISO were 1.14±0.15 Hz (glass), 1.84±0.15 Hz (PDMS 1:20), and 1.54±0.11 Hz (PDMS 1:40). Statistical analysis showed significant differences at t = 20 minutes between the groups (p<0.01). B. Effects of parasympathetic stimulation by addition of 1 μM of ACh. The rates of contraction at t = 1 minute after the addition of ACh were 0.56±0.07 Hz (glass), 0.51±0.10 Hz (PDMS 1:20), and 0.63±0.12 Hz (PDMS 1:40); consequently, no significant differences were observed at t = 1 minute between the groups (p = 0.700). The rates of contraction at t = 20 minutes after the addition of ACh were 0.52±0.12 Hz (glass), 0.45±0.11 Hz (PDMS 1:20), and 0.69±0.10 Hz (PDMS 1:40); no significant differences were observed at t = 20 minutes between the groups (p = 0.33). C. No changes in σ_period_ were observed with time for cardiomyocytes cultivated on glass after the addition of ISO. The σ_period_ for cardiomyocytes cultivated on PDMS 1:20 was decreased significantly 10, 15, and 20 minutes after the addition of ISO (p<0.05, Mann-Whitney comparison test). There was a tendency towards a decreased σ_period_ for cardiomyocytes cultivated on PDMS 1:40 in the presence of ISO. D. A significant increase in σ_period_ at t = 1 minute (p<0.05, Mann-Whitney comparison test) was observed for cardiomyocytes cultivated on glass after the addition of ACh. No significant change was observed for the remaining measurements (t = 5, 10, 15, and 20 minutes). The σ_period_ for cardiomyocytes cultivated on PDMS 1:20 only increased significantly at t = 10 and 15 minutes after the addition of ISO (p<0.05, Mann-Whitney comparison test). A significant increase in σ_period_ was observed for PDMS 1:40 at t = 1, 5, 10, 15, and 20 minutes (p<0.05, Mann-Whitney comparison test).

Fig 5A shows the time-dependent effect of sympathetic stimulation by exposure to 100 nM of ISO. The frequency of contractions at t = 1 minute after the addition of ISO increased by 61.9% (glass), 64.2% (PDMS 1:20), and 55.9% (PDMS 1:40). Interestingly, β-adrenergic stimulation appears to be transient in cardiomyocytes cultured on glass as there was a small increase of 1.3% at t = 20 minutes. However, ISO effects were significantly maintained over the same period in monolayers cultured on PDMS (84.0% with PDMS 1:20 and 53.6% with PDMS 1:40 vs. glass, p<0.05).

Fig 5B shows the effect of parasympathetic stimulation with 1 μM of ACh that resulted in slower spontaneous activity. Over a 20 minutes period of acquisition after adding ACh, the greatest observed slowing of spontaneous activity was 49%, 61%, and 57% for glass, PDMS 1:20, and PDMS 1:40, respectively. The rate of contractions at t = 1 minute after the addition of ACh was decreased by 43.5% (glass), 49.2% (PDMS 1:20), and 37.0% (PDMS 1:40). Consequently, no significant differences were observed at t = 1 minute between the groups (p = 0.7). The frequency of contractions at t = 20 minutes after the addition of ACh was not different between the groups (p = 0.33), as it was decreased by 47.6% (glass), 55.3% (PDMS 1:20), and 30.6% (PDMS 1:40).

The number of pauses (duration longer than 3 seconds) for the set of samples was estimated pre-drug and at t = 1 minute post-drug. The results of this analysis can be found in S7A Fig (pauses for ISO study) and S7B Fig (pauses for ACh study). For ISO, the most interesting result is the tendency towards the greatest decrease in the number of pauses in the PDMS 1:20 group (comparison between pre and post-ISO). This result is in accordance with decreased σ_period_ presented in Fig 5C. As for the effects of ACh, all substrates show an increased number of pauses, although only the PDMS 1:40 group shows a significant augmentation (post-ACh vs. pre-ACh comparison).

### Parasympathetic and sympathetic stimulation: effect on instability of spontaneous activity

This section focuses on the impact of parasympathetic or sympathetic stimulation on the instability of spontaneous activity. Mean σ_period_ values for the control group on glass were 0.19*±*0.04 second (pre-ISO), 0.15*±*0.03 second (t = 1 minute), 0.26*±*0.06 second (t = 5), 0.15*±*0.03 second (t = 10), 0.23*±*0.07 second (t = 15), and 0.21*±*0.06 second (t = 20). Mean σ_period_ values for the control group on PDMS 1:20 were 0.39*±*0.07 second (pre-ISO), 0.21*±*0.05 second (t = 1), 0.19*±*0.04 second (t = 5), 0.15*±* 0.03 second (t = 10), 0.11*±*0.02 second (t = 15), and 0.15*±*0.03 second (t = 20). For cultures on PDMS 1:20, σ_period_ was decreased significantly up to 20 minutes after the addition of ISO (p<0.05, Mann-Whitney comparison test). There was a tendency for mean σ_period_ values in the PDMS 1:40 control group to decrease; values were 0.25±0.05 second (pre-ISO), 0.19*±*0.04 second (t = 1), 0.22*±*0.05 second (t = 5), 0.19*±*0.04 second (t = 10), 0.12*±*0.03 second (t = 15), and 0.13*±*0.03 second (t = 20). No significant change in σ_period_ was found over time in cardiomyocytes cultured on glass after the addition of ISO over 20 minutes (Fig 5C).

Statistical differences for σ_period_ in the presence of ISO were achieved on PDMS 1:20 after 10 minutes of ISO addition, indicating a stronger effect on this substrate that also had the highest median of σ_period_ (as shown previously in Fig 2A).

Fig 5D illustrates the increase in σ_period_ in cardiomyocyte monolayers after the addition of ACh. Mean σ_period_ values for the ACh group on glass were 0.15±0.04 second (pre-ACh), 0.93*±*0.29 second (t = 1 minute), 1.09*±*0.50 second (t = 5), 1.11*±*0.49 second (t = 10), 1.00*±*0.4*2* second (t = 15), and 0.62*±*0.36 second (t = 20). A significant increase in σ_period_ between pre-ACh and post-ACh was observed at t = 1 minute (p<0.05, Mann-Whitney comparison test), while no significant change was observed for the remaining measures (t = 5, 10, 15, and 20 minutes) on glass cultured monolayers. For the ACh group on PDMS 1:20, mean σ_period_ values were 0.35*±*0.08 second (pre-ACh), 0.36*±*0.10 second (t = 1 minute), 1.30*±*0.44 seconds (t = 5), 1.25*±*0.38 seconds (t = 10), 1.91*±*0.63 seconds (t = 15), and 0.77*±*0.29 second (t = 20). For cardiomyocytes cultured on PDMS 1:20, σ_period_ was increased significantly pre-ACh vs. post-ACh at t = 5 and 10 minutes after the addition of ACh (p<0.05, Mann-Whitney comparison test). For the ACh group on PDMS 1:40, mean σ_period_ values were 0.27*±*0.06 second (pre-ACh), 1.07*±*0.30 seconds (t = 1 minute), 1.45*±*0.37 seconds (t = 5), 1.83*±*0.46 seconds (t = 10), 1.71*±*0.43 seconds (t = 15), and 1.68*±*0.42 seconds (t = 20). According to these results, a significant increase in σ_period_ between pre-ACh and post-ACh was observed for PDMS 1:40 at t = 1, 5, 10, 15 (p<0.05, Mann-Whitney comparison test), and 20 minutes (p<0.01, Mann-Whitney comparison test).

Interestingly, increased σ_period_ could be observed at t = 1 minute after the addition of ACh for glass and PDMS 1:40 substrates, while the effect on PDMS 1:20 appeared later at t = 5 minutes. Overall, this indicates that the substrate influenced drug action on spontaneous activity. In this example, the destabilization effect of ACh seems to be decreased when cardiomyocytes are cultured on PDMS 1:20.

### Parasympathetic and sympathetic stimulation: effect on 1^st^ activation site

As the number of activation sites is significantly higher on PDMS substrates compared to glass substrate, we checked whether the significant decrease in σ_period_ following ISO injection is associated with a diminution in the number of activation sites.

**Fig 6:**
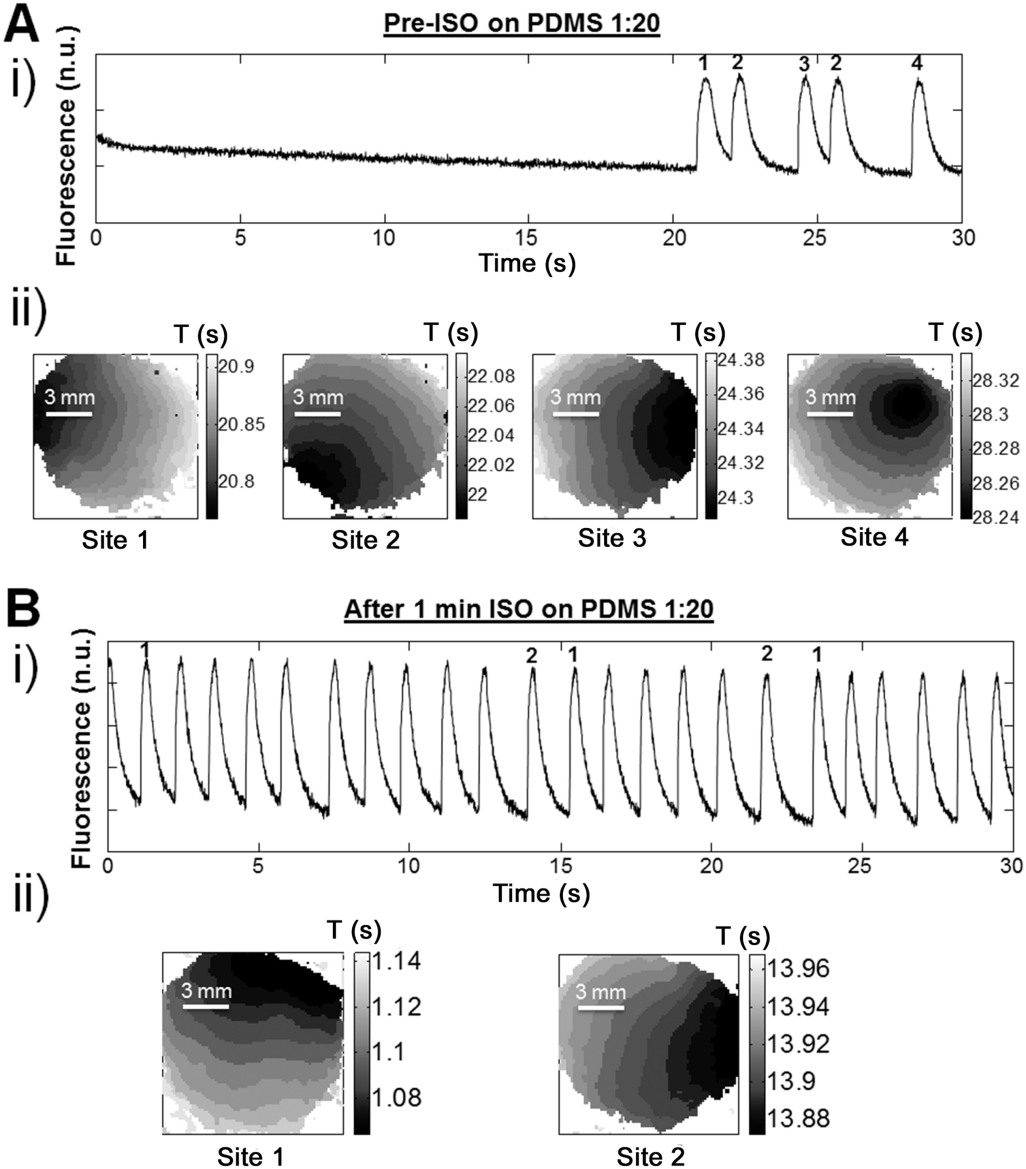
ISO decreases σ_period_ for cardiomyocytes cultivated on PDMS 1:20 and tends to stabilize the rate of contraction. Examples of cardiomyocyte activity stabilization by the addition of isoproterenol. i) A trace of the contractile activity is shown with ii) activation maps of the first beat for each different activation site. **A**. Conditions before the addition of isoproterenol (Pre-ISO) on PDMS 1:20. **B**. One minute after adding ISO (100 nM) on PDMS 1:20 substrates. Pharmacological sympathetic stimulation appears to decrease the number of activation sites (from 4 sites pre-ISO to 2 sites after ISO).

Mapping experiments in presence of ISO show a decrease in the number of activation sites on glass (2 sites pre-ISO compared to 1 site after ISO; S5A and S5B Fig) and PDMS 1:20 (4 sites pre-ISO compared to 2 sites post-ISO; Fig 6A and 6B). However, no change was observed for PDMS 1:40 (3 sites pre-ISO and post-ISO; S5C and S5D Fig). Once more, ISO has greatest effect on PDMS 1:20, which is in agreement with the data showing the effect of ISO on frequency.

Pharmacological parasympathetic stimulation by ACh increased the number of activation sites on glass (2 sites pre-ACh compared to 3 sites post-ACh; S6A and S6B Fig). No change was observed on PDMS 1:20 (2 sites were observed both pre-ACh in S6C Fig and post-ACh in S6D Fig). Mapping cultures on PDMS 1:40 revealed an increase in the number of activation sites (3 sites pre-ACh compared to 2 sites post-ACh, S6E and S6F Fig). Overall, parasympathetic stimulation with ACh did not exert a clear effect on the number of activation site.

### mRNA levels related to the effects of parasympathetic and sympathetic stimulation

There were no significant differences in the mRNA expression of proteins related to parasympathetic (IKACh, Kir 3.1, and Kir3.4; S4D and S4E Fig) or to sympathetic (β1 adrenergic receptors; S4F Fig) stimulation between substrates.

### Stability of cell populations between substrates in culture

Myofibroblasts can be found in cardiomyocyte cultures. These cells can be electrically coupled to cardiomyocytes in culture and possibly in tissue thus affecting depolarization, ectopic activity, electrical conduction, and reentry sensitivity [57–60]. As such, evaluation of possible changes in myofibroblast population between the groups under study has been done. Expression of alpha-actinin gene in primary cultures was evaluated with qPCR (S8 Fig). A similar level of alpha-actinin gene expression was found between the glass and PDMS groups. There is no indication of a difference in the number of cardiomyocytes within monolayers between the groups investigated. Results obtained for alpha smooth muscle actin (alpha-SMA), a marker of myofibroblast, are presented in S9 Fig No significant differences in gene expression of alpha-SMA was found between the groups of interest (glass, PDMS 1:20, PDMS 1:40). These results indicate that no important difference in the cell population could behind the difference in dynamics between glass and PDMS substrates.

## Discussion

To study stretch-related effects including simulating pathological conditions like hypertrophy, pressure-overload [61], or atrial cardiomyocyte remodeling [62], a flexible cell culture substrate like StageFlexer Membrane with Flexcell systems (*Flexcell International Corporation*, *Hillsborough*, *NC*, *USA*) or similar silicone-based membrane (e.g. non-reinforced vulcanized silicone membrane) is generally used [63–65]. We hypothesized that culturing on such elastomeric substrates affects the spontaneous activity of cultured neonatal rat ventricular cardiomyocyte (NRVM) monolayers, based on published studies illustrating the effect of the substrate properties on cardiomyocyte shape [41, 66, 67], calcium transient morphology [21], and spontaneous frequency of contraction (using other types of substrates) [35, 68]. To the best of our knowledge, this is the first investigation aimed at elucidating the impact of silicone-based substrate properties on the spontaneous activity of NRVM. PDMS has numerous advantages as a material for cell study, including optical clarity, biocompatibility, high tolerance to stretch compared to hydrogels, and possibility to modulate its stiffness to reach physiologically relevant ranges [69]. Control of the final geometry is also possible using a simple molding technique for curing the PDMS. This technical aspect is highly important in tissue engineering to control the seeding area and/or apply patterning to the seeding surface to induce orientation of the cells [3]. Culture of cardiomyocytes on a stiff substrate has been shown to impact their function both for force generation [19, 21, 23] and spontaneous activation [35, 68].

Single [70, 71] and multiarmed reentry [72] can be created by point electrical stimulation or spontaneously arise [73] in cardiac monolayers. In the present study, an increased number of spontaneous reentrant events were observed when cardiomyocytes were cultured on a glass substrate compared to a PDMS substrate. This finding increased the intragroup variability. By removing these reentrant events (Fig 1), we obtained a smaller intergroup variability of frequencies. Stiffness differences between glass and PDMS substrates do not significantly affect the spontaneous beating frequency (S2 Fig), but seem to affect the incidence of reentrant activities.

Another factor that could impact spontaneous activity of monolayers is cell density [74]. Density influences cardiomyocyte function because cells react to the substrate and also to their neighboring cells through mechanosensitivity [75]. In our study, we tried to limit the impact of cell density by counting living cells before seeding and by plating the same quantity of cells on each substrate. Thus, the significant increase in the number of nuclei found for PDMS 1:20 (S3 Fig) indicates a possibly greater proliferation on this substrate. In cases of initial low density at seeding, increased cell proliferation could lead to stronger intercellular coupling and favor faster spontaneous frequency [74]; however, we did not observe this effect.

### Stability of the spontaneous activity

Our study illustrated an increased temporal variability in the interbeat period (σ_period_) on PDMS 1:20 (Fig 2). Lower expression of Cx43 tends to uncouple cardiac cells and could highlight the intrinsic rhythm of each cell (pacemaker activity) [55]. Subsequently, the down regulation of Cx43 mRNA detected in our study could explain why we observed an increased number of activation sites on PDMS compared to the glass substrate (Fig 3). Reentry is more likely to occur in excitable tissue that is partially uncoupled [1]. Probability of reentrant activity has been shown to be increased in low to mildly coupled monolayers [54,72]. Here, Cx43 mRNA has the tendency to be lower on PDMS 1:20 compared to glass while the incidence of reentry is increased on glass compared to PDMS. This result indicates that other factors could protect from spontaneous formation of reentry on PDMS. This might be explained by a non-uniform distribution of Cx as highlighted by the change in local activation delay along the trajectory (more importantly for the region close to 2π illustrated in Fig 1). A non-uniform distribution of Cx can facilitate reentry and non-uniform conduction properties [76]. However, curvature effects can also induce change in conduction velocity [77, 78]. The increase in the number of activation sites on PDMS could possibly be linked to the increased median σ_period_ calculated in PDMS compared to glass (52.6% for PDMS 1:20 and 21.1% for PDMS 1:40). These different σ_period_ values are affected by the increased variation between beats, but also by occasional pauses between bursts of faster beats in PDMS 1:20 recordings. These results are in agreement with Ponard *et al*, who demonstrated that stochastic gating of transmembrane currents and calcium release channels can affect ion channel turnover and thereby induce variability in the spontaneous rate [79]. However, we detected a larger change in spatial position of focal activation than Ponard *et al*., which we attribute to greater distances (~6 mm) between interacting pacemaker sites compared with smaller distances (1 mm) reported by the authors [79].

### Changes in the voltage clock—associated mRNA and effects on spontaneous activity

The voltage clock encompasses membrane currents that play a role in the spontaneous activity of cardiomyocytes, and more importantly, in sinus node cells [10]. Modifications in expression or function of membrane currents could alter the rate of spontaneous activity. In our study, we observed an increased expression of CaV3.1 (I_CaT_) and HCN2 (I_f_) mRNA. Increased expression of Ca^2+^-permeable (T- and L-type) channels and HCN channels including HCN2 [80] could promote automaticity [8, 11, 81, 82]. Although not a significant finding, the tendency towards decreased Kir2.1 expression (Fig 4) could also favor acceleration of spontaneous activity [83]. However, no significant difference in frequency was detected (S2 Fig). Instead, there was a significant increase in the number of activation sites on PDMS (Fig 3). One possible explanation is that the global change in mRNA expression could lead to protein changes that do not cause a linear shift in the local intrinsic period of individual spontaneous cells. We perceive monolayers to consist of quiescent and spontaneous cells (with a set of individual frequencies) [55]. Cells with higher frequency are thought to become the drivers of monolayers; however, locally connected strong pacemakers could more easily overcome the source/sink ratio and drive spontaneous activation. Therefore, a decrease in cellular coupling (through Cx43 mRNA) and increase in local spontaneous activity (decreased Kir2.1 and increased HCN2) would favor regionalized depolarization. As such, these results suggest that culture on a PDMS substrate may favor the appearance of spontaneous activity in multiple cells and increase the number of regions that can depolarize and generate a propagating response.

### Response of different substrates to sympathetic and parasympathetic stimulation

Vagal nerve stimulation slows heart rate, predominantly by the action of the vagal neurotransmitter, acetylcholine, at muscarinic receptors. The binding leads to the dissociation of G proteins and activation of the potassium current, I_K,Ach_ [84]. In our study, ACh (1 μM) significantly decreased the rate of spontaneous activity of monolayers in all groups, with the greatest decrease on PDMS 1:20 compared to the glass substrate (Fig 5B) and the greatest instability on PDMS (Fig 5D, higher number of time points with significant increase in σ_period_ compared to pre-ACh data). Increased instability is a known hallmark of ACh-induced slowing effects [85]. Interestingly, instability was more rapid on glass and PDMS 1:40, while its effect was more gradual on PDMS 1:20. Increased σ_period_ was only visible after 5 minutes on PDMS 1:20. The mRNA levels for both Kir3.1 and Kir3.4 (S4D and S4F Fig), coding for ACh-activated K+ channels, were not significantly different but showed a tendency towards a slight increase on PDMS. At early developmental stages, I_K,ACh_ is known to be primarily formed by Kir3.1, while in late embryonic and adult cells, Kir3.4 is the predominant subunit [86]. Here, the change in culture substrate did not induce variation in the ratio of Kir3.4/ Kir3.1, indicating that there was no modification in the rectification properties of I_K,ACh_ and therefore function between substrates.

It has recently been shown that substrate stiffness can regulate cellular responses to β-adrenergic receptor agonist (ISO) in human mesenchymal stem cells [87]. It was unknown whether ISO could exert different effects on cardiomyocytes cultured on PDMS. Interestingly, cardiomyocytes cultured on PDMS (1:20) exhibited the greatest increase in frequency of spontaneous activity by 100 nM ISO (relative to pre-drug at t = 0). More precisely, the effect lasted significantly longer on PDMS 1:20 compared to culturing on glass. ISO significantly reduced σ_period_ on the PDMS 1:20 substrate (Fig 5C), which confirms that instabilities are greater for slower activity [85]. However, we found that decrease in instability is also associated with a diminution in the number of activation sites (Fig 6 and S5 Fig). This result is in agreement with a previous study that claims the positive inotropic effect of ISO is associated with a decrease in the relative value of the potentiating effect of the pause (instability) [88].

We hypothesized that expression of beta-1 adrenergic receptor (AdrB1) could be altered by the culture substrate; however, no significant change at the mRNA level was found. It remains to be determined if post-transcriptional regulation process including a change in internalization rate of the receptor [87] could be responsible for the differences. The increased duration of the effect of ISO stimulation may be a consequence of the substrate on phosphodiesterase action. cAMP plays a central role in regulating metabolic and cellular processes through protein kinase A (PKA). Phosphodiesterases are directly involved in the degradation of cAMP controlling the duration and intensity of the response in cAMP signaling [89] and has an impact on sinoatrial node activation frequency [90].

## Conclusion

This study is part of the greater project aimed at the development of a system to evaluate the effects of stretch on cardiomyocytes. To apply deformation, a flexible substrate is needed. Due to the brittle characteristics of currently available hydrogels, we opted for the use of a deformable silicone-based substrate to approach *in vivo* tissue stiffness. According to our results, selecting the appropriate material for the substrate is highly important since it will affect the *in vitro* spontaneous activity via ionic channel expression modulation. Our study also presents evidence that the substrate mechanical properties can influence the sensitivity to sympathetic (ISO) and parasympathetic (ACh) stimulation, and act as a stabilizer for the propagation of activation through changes in Cx43 mRNA. These results reveal the importance of carefully selecting the culture substrate involving mechanical stimulation, especially for tissue engineering investigations or pharmacological high-throughput screenings of cardiac tissue analog.

## Supporting Information

S1 Appendix Additional methodology for myofibroblast population evaluation. (DOCX)

S2 Appendix Young’s modulus equation. (DOCX)

S1 Fig Stiffness of PDMS with different ratios of curing:base agents. To determine Young’s modulus, the PDMS substrate was molded in a cylindrical mold with defined width and length. Molds were cured at 37°C for 48 hours. Young’s modulus was calculated as the slope of the stress/strain curve that was created using a set of weights ranging from 10 to 150 g (except for PDMS 1:40 where the maximum weight was 30 g). Attempts were made to remain within the limits of linear elasticity. PDMS was mixed in ratios of 1:5, 1:10, 1:15, 1:20, 1:25, 1:30, and 1:40 to produce substrates with mean moduli of 974±32 kPa, 293±8 kPa, 112±6 kPa, 87±22 kPa, 42±6 kPa, 27±4 kPa, and 16±4 kPa, respectively. Inset: low modulus values for large-mixed ratios.
(TIF)

S2 Fig Rate of spontaneous activity of cardiomyocytes after 48 hours culture. Mean spontaneous activity of cardiomyocytes after 48 hours culture was 1.34±0.25 Hz (glass), 1.0±0.08 Hz (PDMS 1:20), and 0.93±0.08 Hz (PDMS 1:40); n = 11. The rate of spontaneous activity tends to decrease when cardiomyocytes are cultivated on PDMS with a greater effect on softer substrates (p = NS). The large error bar for the mean spontaneous frequency measured on glass can be explained by a group of data with high-frequency rate that match the rate of reentry imaged in the calcium mapping experiments (**A**). After removing the data with frequency greater than 3 Hz assumed to be reentrant activity, mean spontaneous activity measured by videomicroscopy on glass is 1.03±0.09 Hz (glass), 1.01±0.08 Hz (PDMS 1:20) and 0.93±0.08 Hz (PDMS 1:40) (**B**).
(TIF)

S3 Fig Number of nuclei. Confocal imaging of neonatal rat cardiomyocytes was performed to determine the number of nuclei in monolayers cultured on different substrates. There was a significant increase in the number of nuclei on the PDMS 1:20 substrate compared to glass and PDMS 1:40 (p = 0.04).
(TIF)

S4 Fig Role of the proteins expression on spontaneous activity. CaV3.2 mRNA expression appears to be lowered when cardiomyocytes were cultivated on PDMS compared to glass (p = NS) (**A**). No change in HCN4 mRNA expression was observed when cardiomyocytes were cultivated on PDMS compared to glass (p = NS) (**B**). Control data (no drug) of spontaneous rate of contraction showing no appreciable differences over time (**C**). No significant changes were observed in mRNA expression of proteins related to parasympathetic (I_K,ACh_, Kir 3.4 (**D**), and Kir3.1 (**E**)) or to sympathetic (β1 adrenergic receptors (**F**)) stimulation.
(TIF)

S5 Fig Effect of ISO on the σ_period_ in monolayer of cardiomyocytes. Conditions before the addition of isoproterenol (Pre-ISO) on glass (**A**). On glass substrates, pharmacological sympathetic stimulation with ISO (100 nM) tends to decrease the number of activation sites after 1 minute (from 2 sites pre-ISO to 1 site after ISO) (**B**). Conditions before the addition of isoproterenol (Pre-ISO) on PDMS 1:40 (**C**). On PDMS 1:40 substrates, pharmacological sympathetic stimulation with ISO (100 nM) did not change the number of activation sites after 1 minute (3 sites for both pre-ISO and post-ISO) (**D**).
(TIF)

S6 Fig Effect of ACh on σ_period_ in cardiomyocyte monolayers. Addition of acetylcholine (ACh) to cardiomyocyte monolayers. i) A trace of contractile activity is shown with ii) activation maps of the first beat for each different activation site. Conditions before the addition of ACh (Pre-ACh) on glass, PDMS 1:20, and PDMS 1:40, respectively (**A, C and E**). On glass substrates, pharmacological parasympathetic stimulation with ACh (1 μM) tends to increase the number of activation sites after 1 minute (from 2 sites pre-ACh to 3 sites after ACh) (**B**). On PDMS 1:20 substrates, ACh (1 μM) stabilized the number of activation sites after 1 minute (2 sites for both after ACh and pre-ACh) (**D**). On PDMS 1:40 substrates, ACh (1 μM) tends to decrease the number of activation sites after 1 minute (from 3 sites pre-ACh to 2 sites after ACh) (**F**).
(TIF)

S7 Fig Number of pauses: influence of the parasympathetic and sympathetic stimulation. The number of pauses over 3 seconds was evaluated. Before and after the addition of ISO (**A**). Before and after the addition of ACh. Pre-drug and post-drug (at t = 1 minute) differences for each substrate (glass, PDMS 1:20, and PDMS 1:40) were compared with a Wilcoxon matched-pairs test (**B**).
(TIF)

S8 Fig Comparison of alpha-actinin mRNA expression. n.s. indicates that the difference is non-significant.
(TIF)

S9 Fig Comparison of alpha-SMA mRNA expression. n.d. indicates that the sample level was under detection limits and n.s. indicates that the difference is non-significant.
(TIF)

S1 Table Primer sequences. (DOCX)
