# Supplementary material for: Spatiotemporal Stability of Neonatal Rat Cardiomyocyte Monolayers Spontaneous Activity Is Dependent on the Culture Substrate
Source: PLoS One. 2015 Jun 2;10(6):e0127977. doi: 10.1371/journal.pone.0127977 (PMC4452796; doi:10.1371/journal.pone.0127977)
Supplement: S1 Appendix — (DOCX) [file pone.0127977.s001.docx]

**S10 Appendix A.1. Additional methodology for myofibroblast population evaluation.**

qPCR was performed as described in the Materials and Methods section with slight modification as the use of a new qPCR system for analysis (StepOnePlus Real-Time PCR System, *Life Technologies*). The geometric mean of the expressions of HPRT was used for normalization. New data were also analyzed with StepOne software v2.3.

Alpha-actinin gene expression was used as a positive control for the cardiomyocyte population (primer sequences: Forward GAGACGGCAGCTAACAGGAT and Reverse CTTGCATGGTCTTCTCCGGT). To evaluate the presence and possible impact of fibroblast/myofibroblast in primary culture experiments, expression levels of alpha smooth muscle actin (alpha-SMA) were evaluated by qPCR (primer sequences: Forward AGCATCCGACCTTGCTAACG and Reverse CACGAAGGAATAGCCACGCT).

A total of 5 groups were tested, which includes the 3 substrates (glass, PDMS 1:20, and PDMS 1:40), and conditions are described in the Materials and Methods section. Freshly dissociated cardiomyocytes (Cardio FD group) that have not been cultured (t=0, after isolation procedures) were added. Cells maintained in culture for 5 days were also isolated, with 1 passage at day 2 to remove the majority of cardiomyocytes and retain an enriched fibroblast/myofibroblast population (Fibro/Myofibro group).
