# Supplementary material for: Spatiotemporal Stability of Neonatal Rat Cardiomyocyte Monolayers Spontaneous Activity Is Dependent on the Culture Substrate
Source: PLoS One. 2015 Jun 2;10(6):e0127977. doi: 10.1371/journal.pone.0127977 (PMC4452796; doi:10.1371/journal.pone.0127977)
Supplement: S2 Appendix — (DOCX) [file pone.0127977.s002.docx]

**S11 Appendix B.1**

_E_ _=_ tensile stress _=_ σ _=_ F/Ao _=_ FLo _(B.1)_

tensile strain Ɛ ΔL/Lo AoΔL

Where:

E is Young's modulus (modulus of elasticity);

F is the force exerted on an object under tension;

A_0_ is the original cross-sectional area through which the force is applied;

ΔL is the amount by which the length of the object changes;

L_0_ is the original length of the object.
