# Supplementary figures and images for: Spatiotemporal Stability of Neonatal Rat Cardiomyocyte Monolayers Spontaneous Activity Is Dependent on the Culture Substrate
Source: PLoS One. 2015 Jun 2;10(6):e0127977. doi: 10.1371/journal.pone.0127977 (PMC4452796; doi:10.1371/journal.pone.0127977)

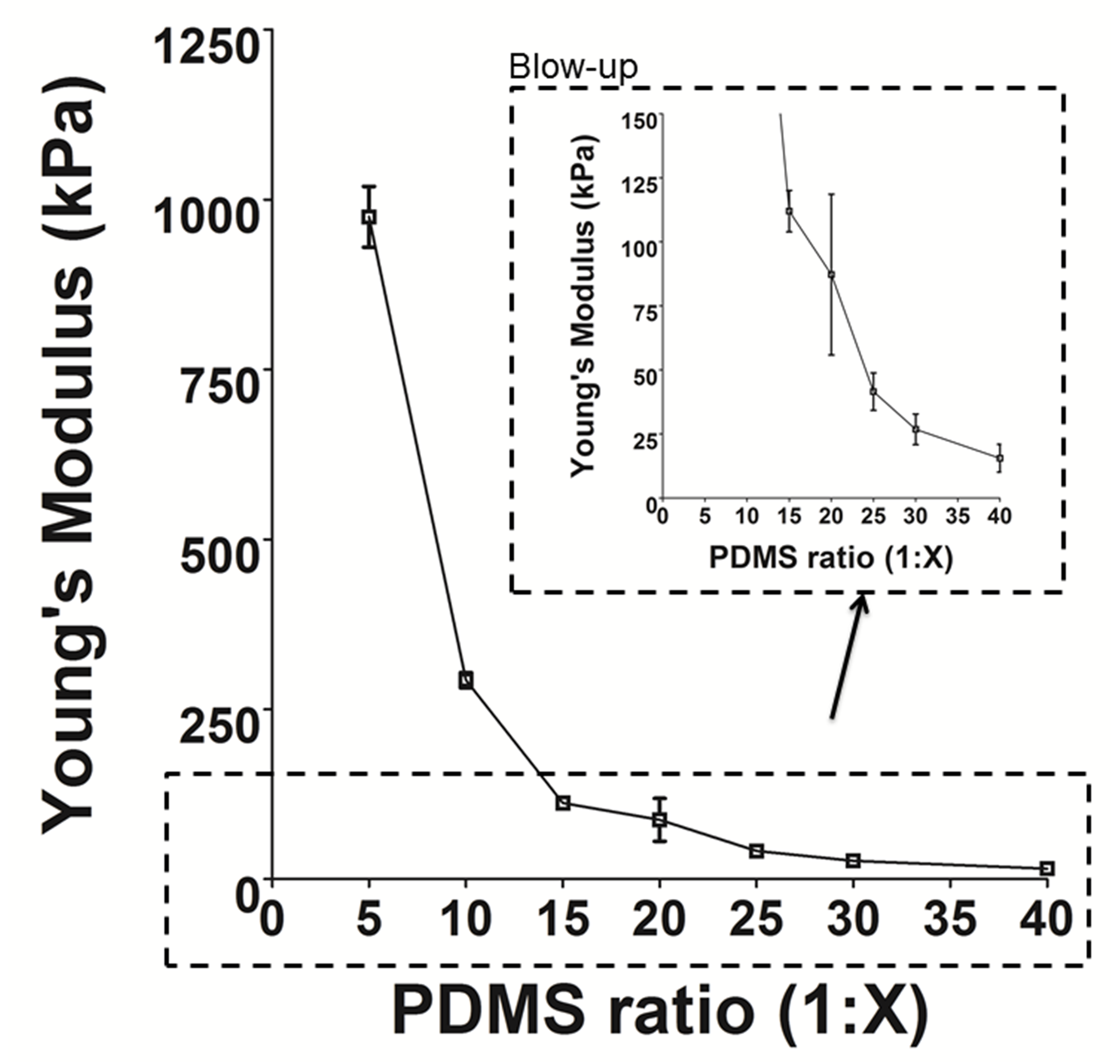

Supplement: S1 Fig — To determine Young’s modulus, the PDMS substrate was molded in a cylindrical mold with defined width and length. Molds were cured at 37°C for 48 hours. Young’s modulus was calculated as the slope of the stress/strain curve that was created using a set of weights ranging from 10 to 150 g (except for PDMS 1:40 where the maximum weight was 30 g). Attempts were made to remain within the limits of linear elasticity. PDMS was mixed in ratios of 1:5, 1:10, 1:15, 1:20, 1:25, 1:30, and 1:40 to produce substrates with mean moduli of 974±32 kPa, 293±8 kPa, 112±6 kPa, 87±22 kPa, 42±6 kPa, 27±4 kPa, and 16±4 kPa, respectively. Inset: low modulus values for large-mixed ratios. (TIF) [file pone.0127977.s003.tif]

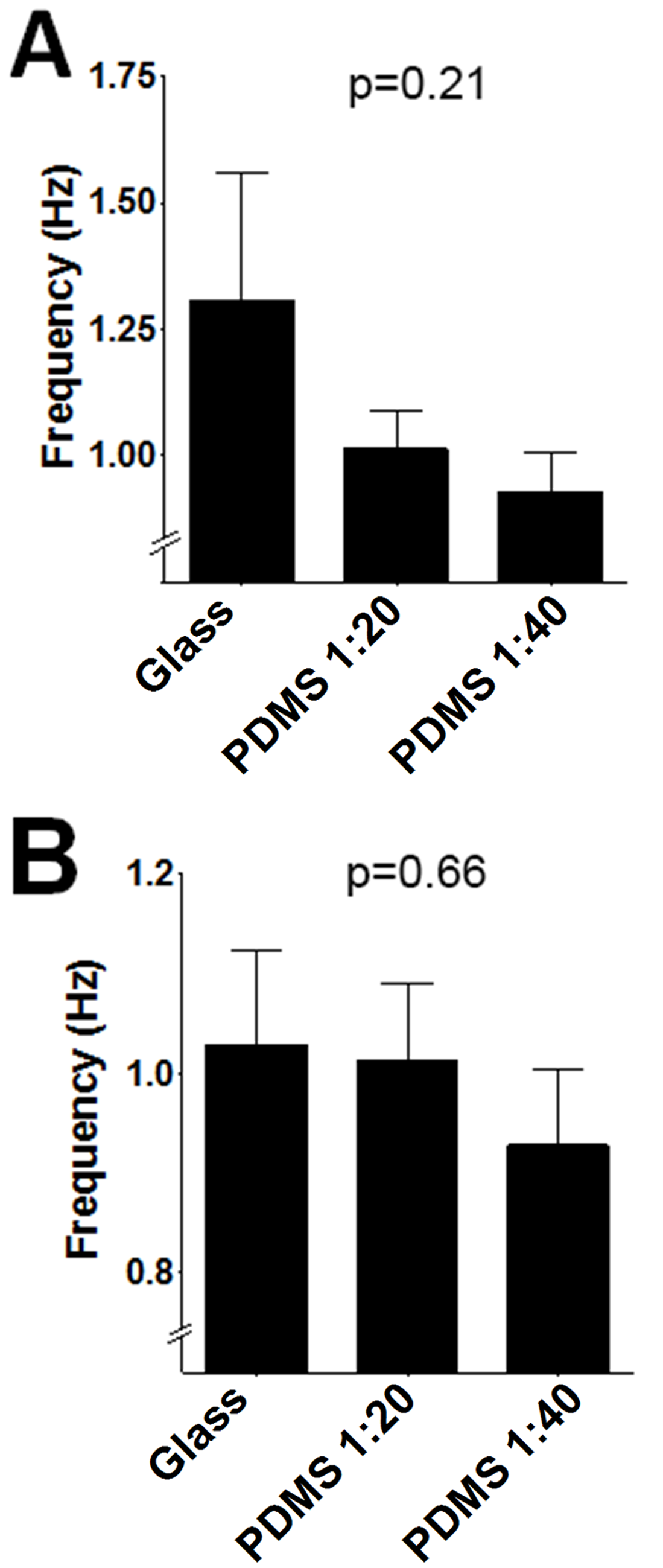

Supplement: S2 Fig — Mean spontaneous activity of cardiomyocytes after 48 hours culture was 1.34±0.25 Hz (glass), 1.0±0.08 Hz (PDMS 1:20), and 0.93±0.08 Hz (PDMS 1:40); n = 11. The rate of spontaneous activity tends to decrease when cardiomyocytes are cultivated on PDMS with a greater effect on softer substrates (p = NS). The large error bar for the mean spontaneous frequency measured on glass can be explained by a group of data with high-frequency rate that match the rate of reentry imaged in the calcium mapping experiments (A). After removing the data with frequency greater than 3 Hz assumed to be reentrant activity, mean spontaneous activity measured by videomicroscopy on glass is 1.03±0.09 Hz (glass), 1.01±0.08 Hz (PDMS 1:20) and 0.93±0.08 Hz (PDMS 1:40) (B). (TIF) [file pone.0127977.s004.tif]

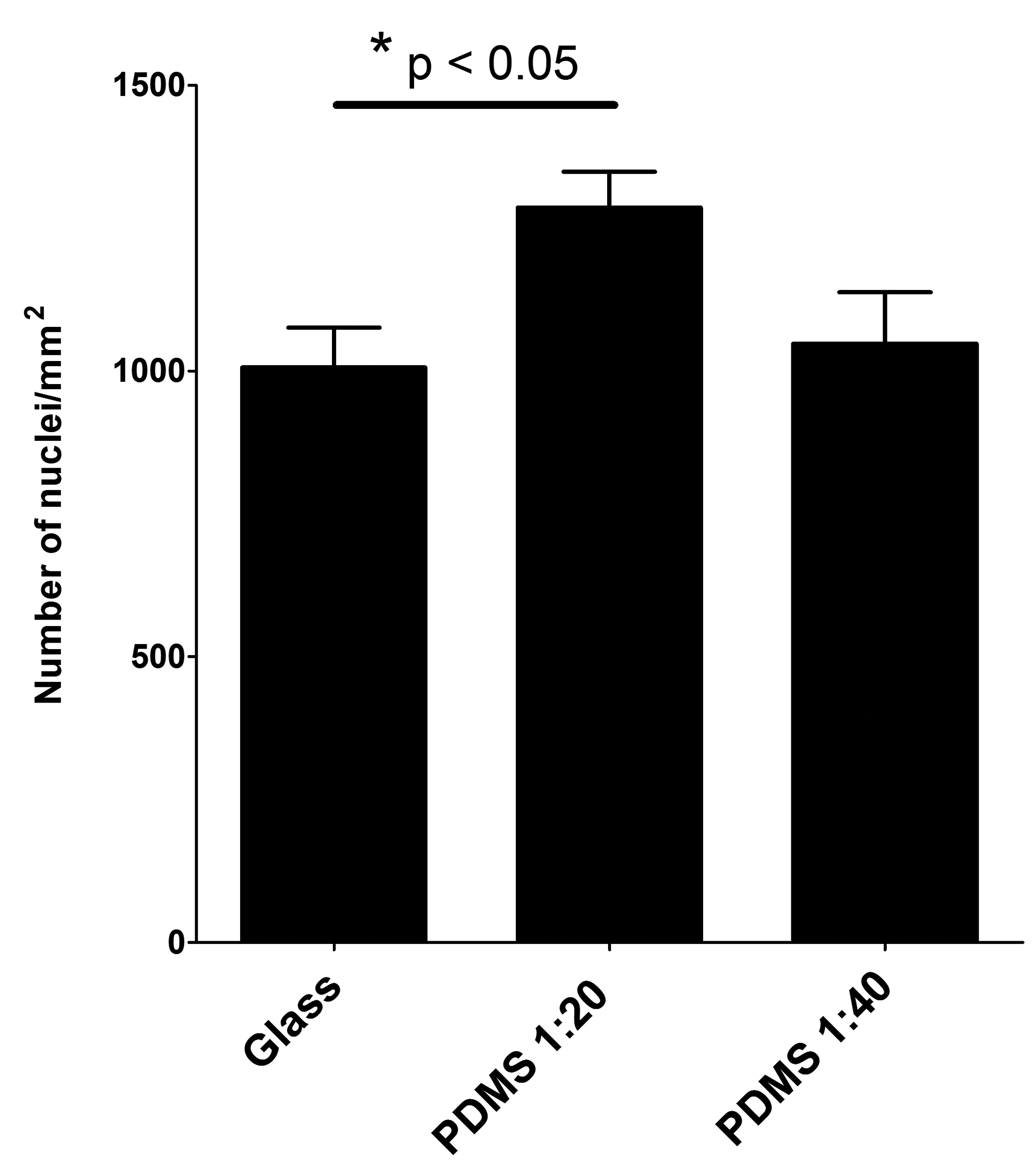

Supplement: S3 Fig — Confocal imaging of neonatal rat cardiomyocytes was performed to determine the number of nuclei in monolayers cultured on different substrates. There was a significant increase in the number of nuclei on the PDMS 1:20 substrate compared to glass and PDMS 1:40 (p = 0.04). (TIF) [file pone.0127977.s005.tif]

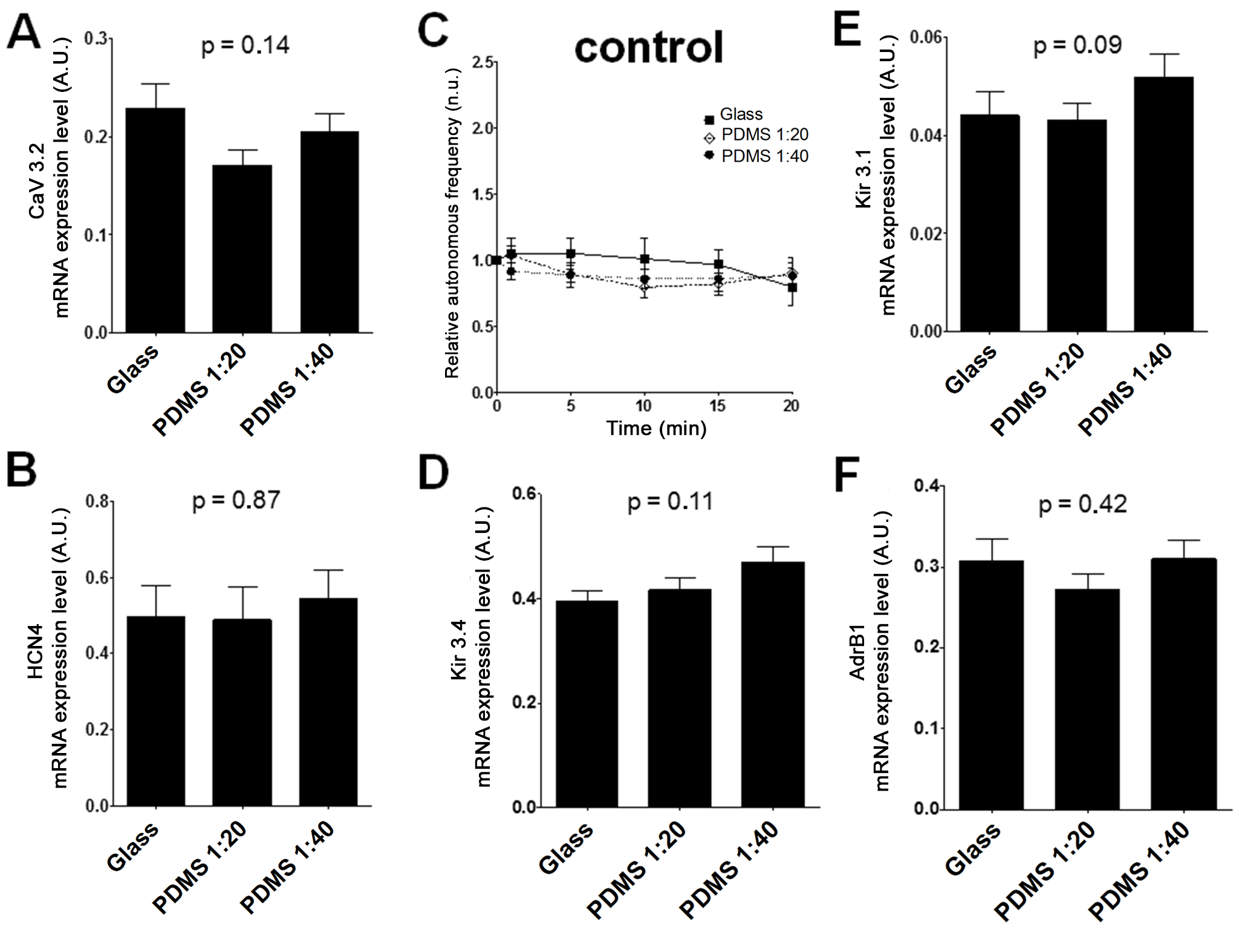

Supplement: S4 Fig — CaV3.2 mRNA expression appears to be lowered when cardiomyocytes were cultivated on PDMS compared to glass (p = NS) (A). No change in HCN4 mRNA expression was observed when cardiomyocytes were cultivated on PDMS compared to glass (p = NS) (B). Control data (no drug) of spontaneous rate of contraction showing no appreciable differences over time (C). No significant changes were observed in mRNA expression of proteins related to parasympathetic (IK,ACh, Kir 3.4 (D), and Kir3.1 (E)) or to sympathetic (β1 adrenergic receptors (F)) stimulation. (TIF) [file pone.0127977.s006.tif]

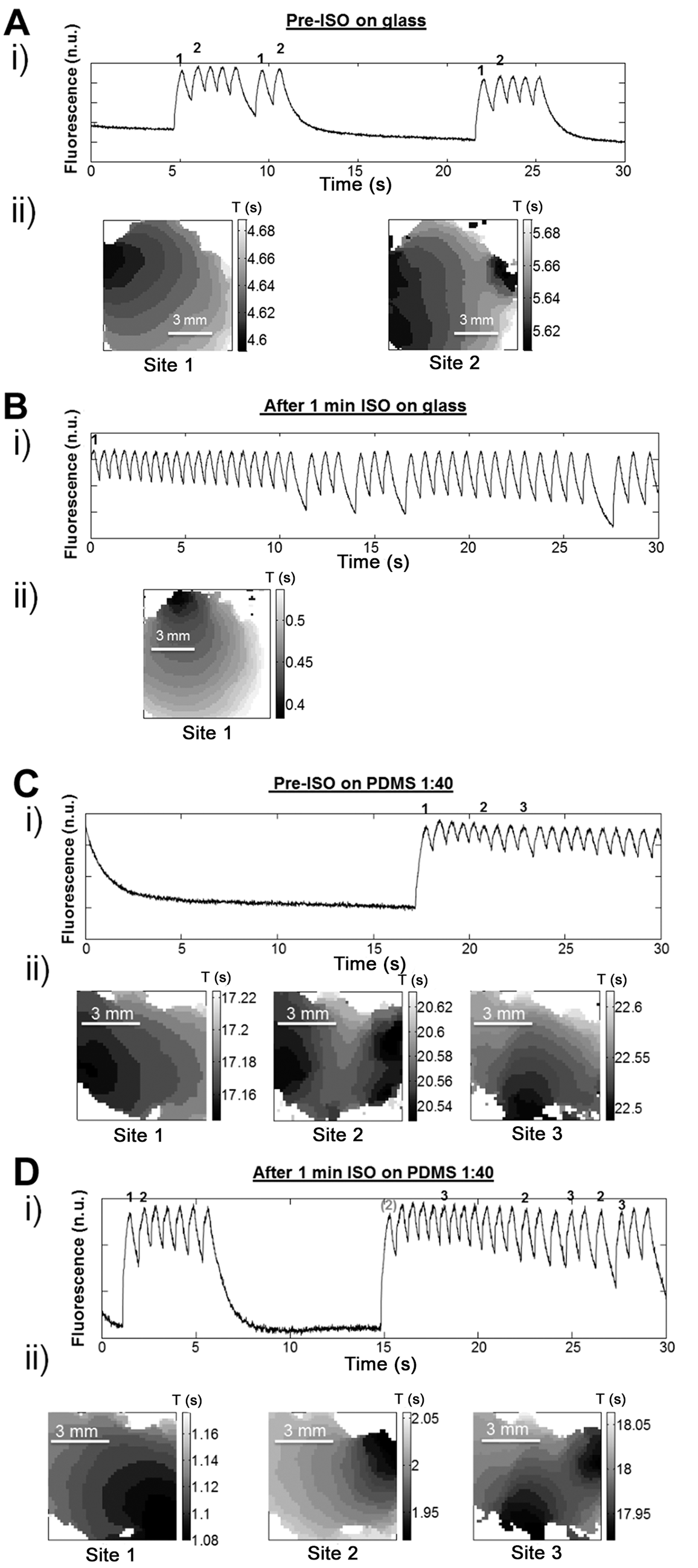

Supplement: S5 Fig — Conditions before the addition of isoproterenol (Pre-ISO) on glass (A). On glass substrates, pharmacological sympathetic stimulation with ISO (100 nM) tends to decrease the number of activation sites after 1 minute (from 2 sites pre-ISO to 1 site after ISO) (B). Conditions before the addition of isoproterenol (Pre-ISO) on PDMS 1:40 (C). On PDMS 1:40 substrates, pharmacological sympathetic stimulation with ISO (100 nM) did not change the number of activation sites after 1 minute (3 sites for both pre-ISO and post-ISO) (D). (TIF) [file pone.0127977.s007.tif]

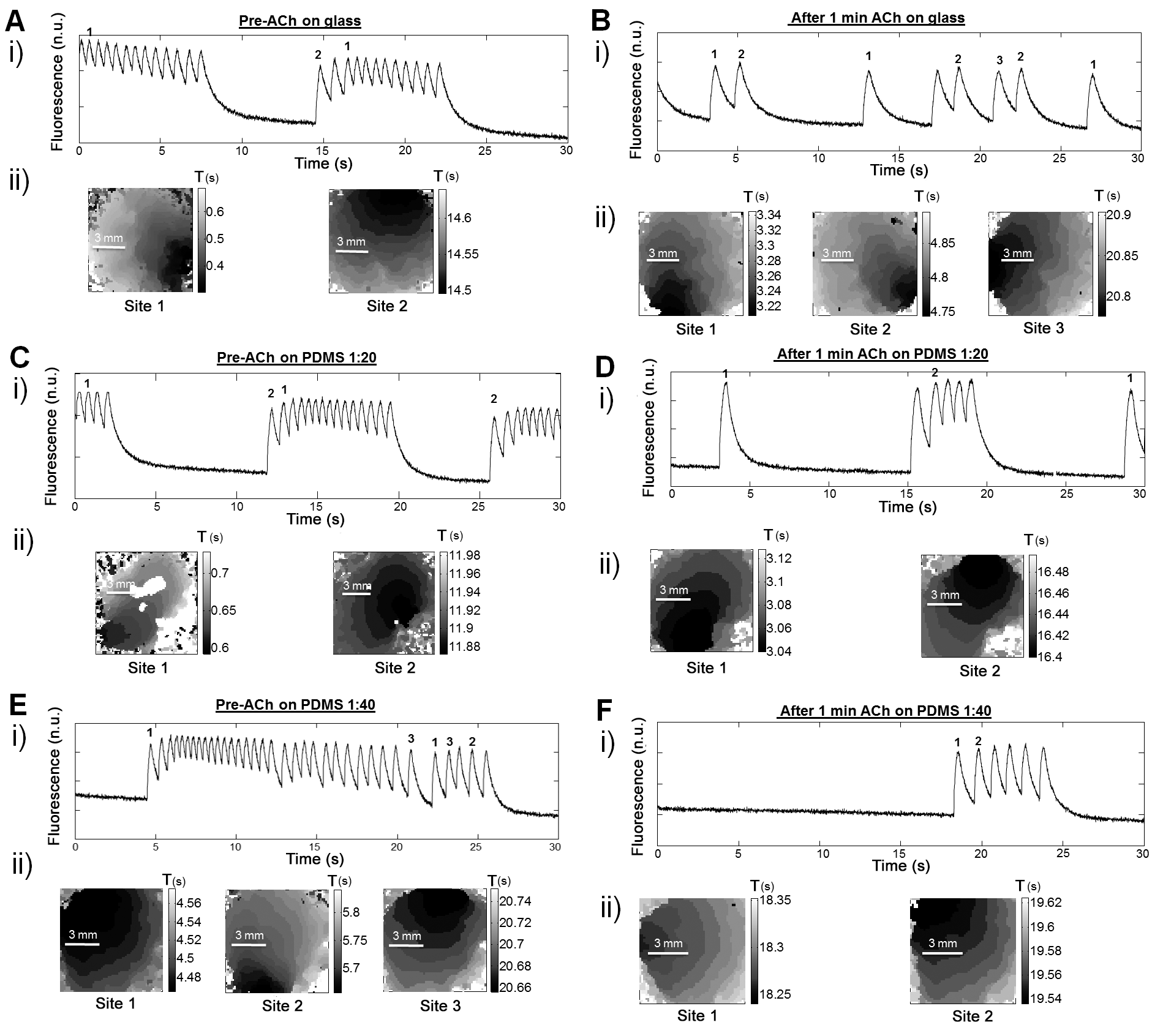

Supplement: S6 Fig — Addition of acetylcholine (ACh) to cardiomyocyte monolayers. i) A trace of contractile activity is shown with ii) activation maps of the first beat for each different activation site. Conditions before the addition of ACh (Pre-ACh) on glass, PDMS 1:20, and PDMS 1:40, respectively (A, C and E). On glass substrates, pharmacological parasympathetic stimulation with ACh (1 μM) tends to increase the number of activation sites after 1 minute (from 2 sites pre-ACh to 3 sites after ACh) (B). On PDMS 1:20 substrates, ACh (1 μM) stabilized the number of activation sites after 1 minute (2 sites for both after ACh and pre-ACh) (D). On PDMS 1:40 substrates, ACh (1 μM) tends to decrease the number of activation sites after 1 minute (from 3 sites pre-ACh to 2 sites after ACh) (F). (TIF) [file pone.0127977.s008.tif]

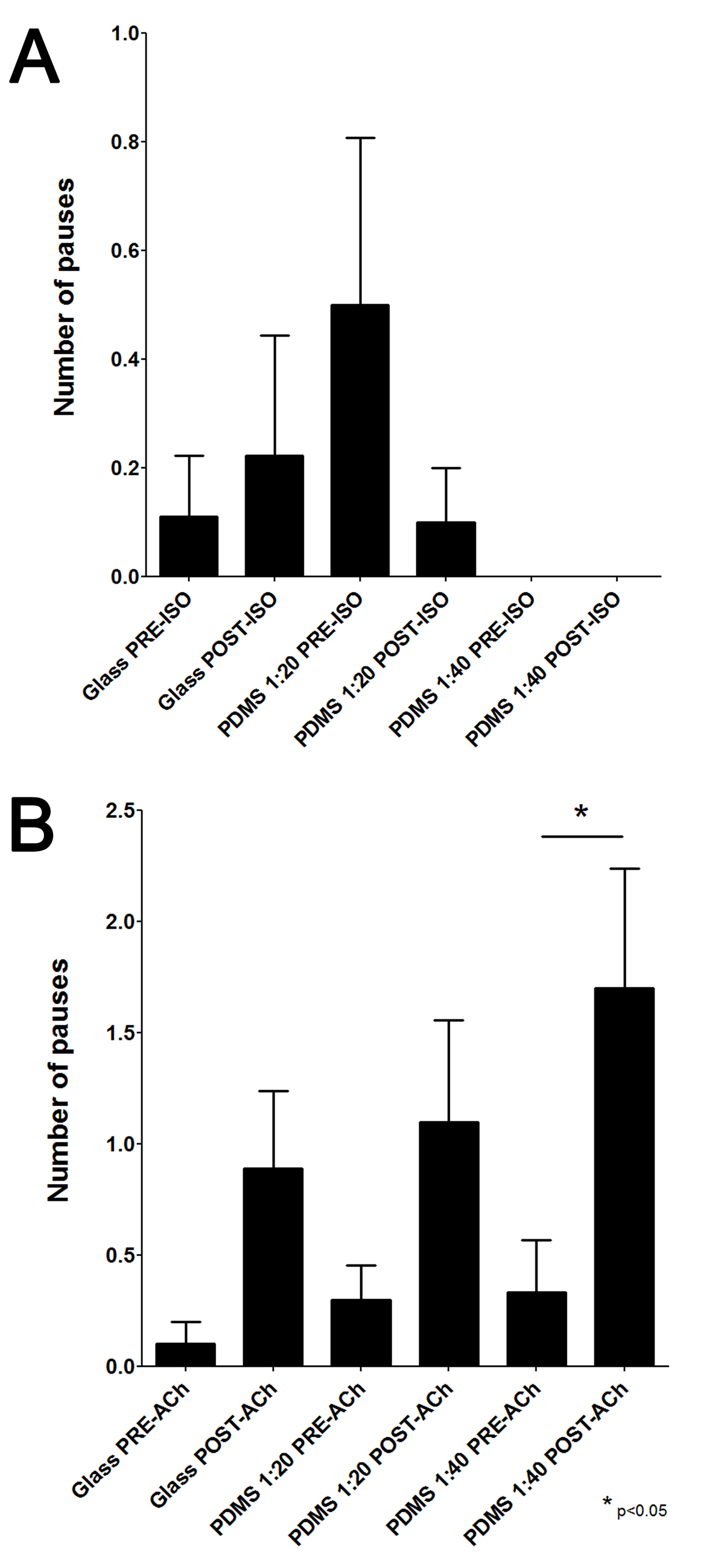

Supplement: S7 Fig — The number of pauses over 3 seconds was evaluated. Before and after the addition of ISO (A). Before and after the addition of ACh. Pre-drug and post-drug (at t = 1 minute) differences for each substrate (glass, PDMS 1:20, and PDMS 1:40) were compared with a Wilcoxon matched-pairs test (B). (TIF) [file pone.0127977.s009.tif]

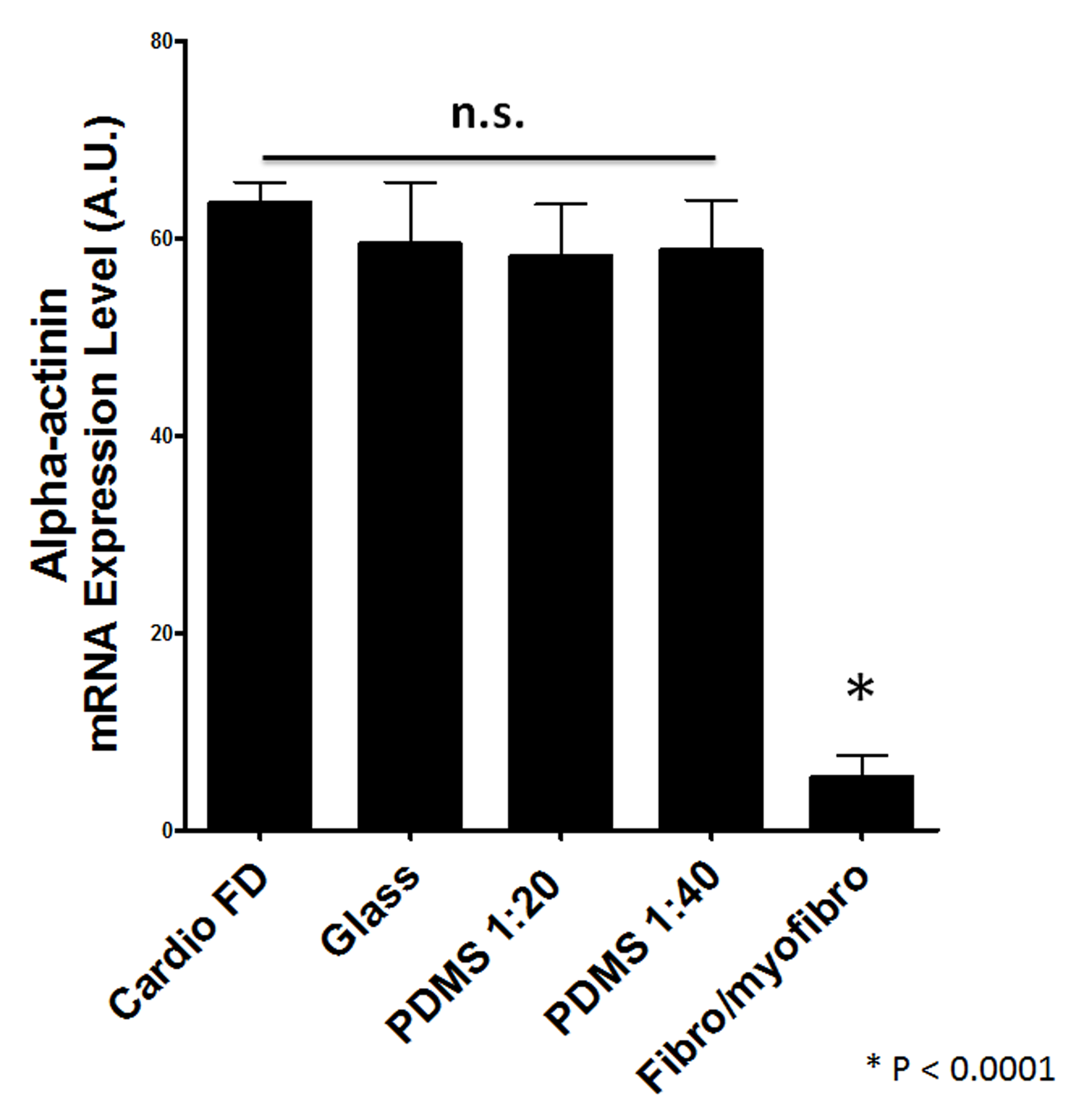

Supplement: S8 Fig — n.s. indicates that the difference is non-significant. (TIF) [file pone.0127977.s010.tif]

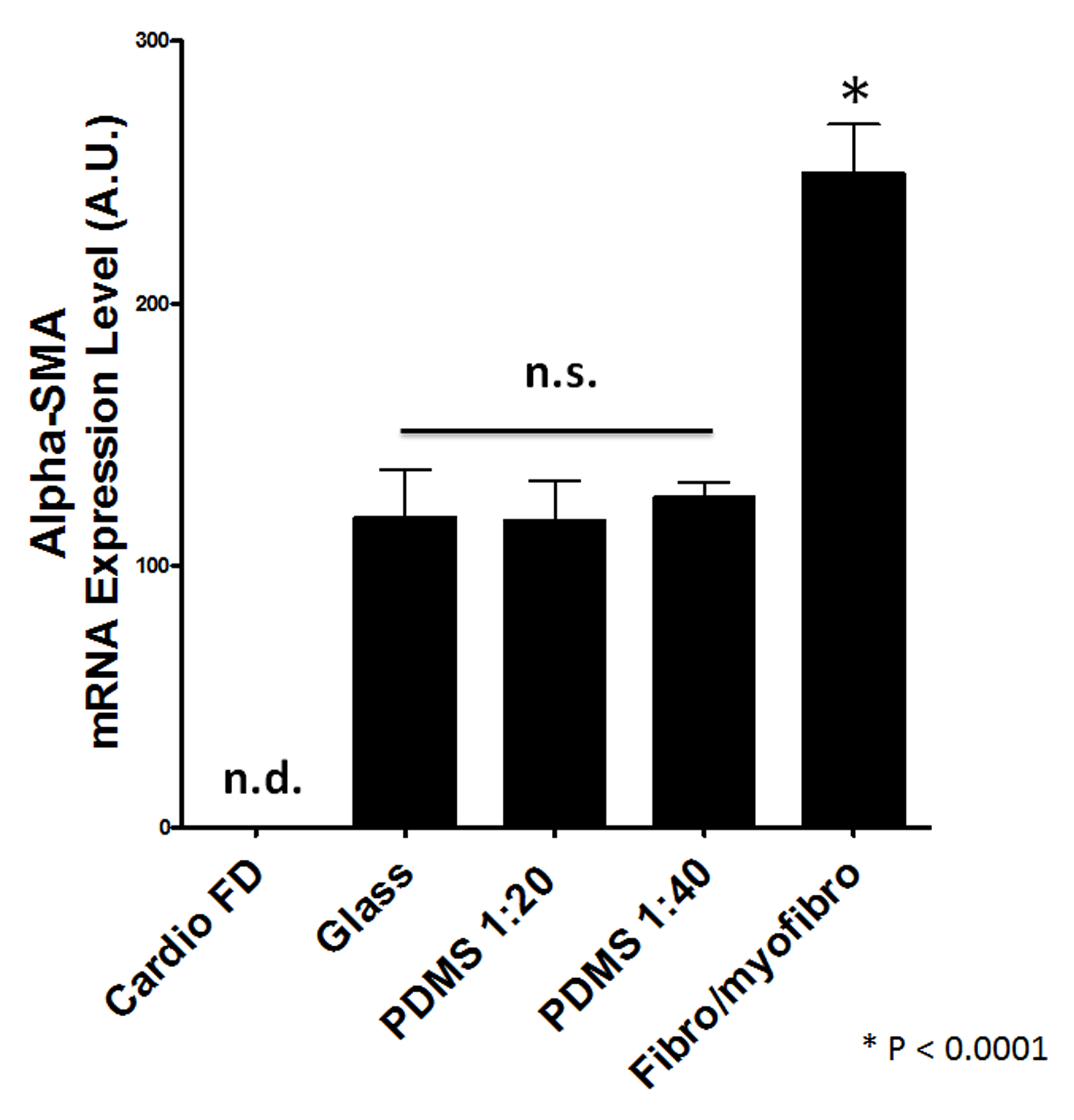

Supplement: S9 Fig — n.d. indicates that the sample level was under detection limits and n.s. indicates that the difference is non-significant. (TIF) [file pone.0127977.s011.tif]
