# Supplementary material for: Spatiotemporal Stability of Neonatal Rat Cardiomyocyte Monolayers Spontaneous Activity Is Dependent on the Culture Substrate
Source: PLoS One. 2015 Jun 2;10(6):e0127977. doi: 10.1371/journal.pone.0127977 (PMC4452796; doi:10.1371/journal.pone.0127977)
Supplement: S1 Table — (DOCX) [file pone.0127977.s012.docx]

**S12 Table**

| \| rAdrb1 Forward \| \| --- \| \| GATCTGGTCATGGGACTGCT \| \| rAdrb1 Reverse \| \| CGTCTACCGAAGTCCAGAGC \| \|  \| \| rHCN4 Forward \| \| GCTTCACGAAGATCCTCAGC \| \| rHCN4 Reverse \| \| ATGCCAATGAGGTTCACGAT \| \|  \| \| rKir2.1 Forward \| \| ACCGCTACAGCATCGTCTCT \| \| rKir2.1 Reverse \| \| GCGAGGCAGAAGATTACCAG \| \|  \| \| rKir3.1 Forward \| \| AGTTCGAGGTGGTCGTCATC \| \| rKir3.1 Reverse \| \| AAAAACGATGACCCCAAAGA \| \|  \| \| rKir3.4 Forward \| \| GAAGTTAGCCCCAAGGGTTC \| \| rKir3.4 Reverse \| \| AGCCTGTTTGGGGATCTTCT \| \|  \| \| rCx43 Forward \| \| CCTTTGACTTCAGCCTCCAA \| \| rCx43 Reverse \| \| CTTGGACCTTGTCCAGAAGC \| \|  \| \| rHCN2 1 Forward \| \| CAATGGGAGGAGATTTTCCA \| \| rHCN2 1 Reverse \| \| AGTCGCTGGGGAAGTCTTG \| \|  \| \| rCav1.2 1 Forward \| \| TCTGCTCTGCCTGACTCTGA \| \| rCav1.2 1 Reverse \| \| CACACAATTGGCAAAAATCG \| \|  \| \| rCav3.1 Forward \| \| GACCTGCAGAACGTCAGCTT \| \| rCav3.1 Reverse \| \| AGCATAGGCAAGGTGTCCAG \| |
| --- | --- | --- | --- | --- | --- | --- | --- | --- | --- | --- | --- | --- | --- | --- | --- | --- | --- | --- | --- | --- | --- | --- | --- | --- | --- | --- | --- | --- | --- | --- | --- | --- | --- | --- | --- | --- | --- | --- | --- | --- | --- | --- | --- | --- |
